# Supplementary material for: Evaluation of a cadaveric wrist motion simulator using marker-based X-ray reconstruction of moving morphology
Source: PeerJ. 2024 May 24;12:e17179. doi: 10.7717/peerj.17179 (PMC11129696; doi:10.7717/peerj.17179)
Supplement: Supplemental Information 1 — The graphs show the wrist angles, tendon forces and actuator displacements during the an average trial from every motion and specimen before (A, C & E) and after (B, D & F) a total wrist replacement. This excludes the radial extension trial of specimen 1, which is included as a representative trial in the main text. For every figure A&B correspond to angles (° ) of the third metacarpal with respect to the radius, represented by rotations about the X (pronation/supination), Y (flexion/extension) and Z (radioulnar deviation) axes of the radial coordinate system. C&D correspond to forces (N) applied to the ECU, ECRB, ECRL, FCU and FCR tendons over the duration of the motion trial. E&F correspond to displacements (mm) of the five linear actuators connected to the tendons over the duration of the motion trial. The mean (s.d.) angle/force/displacement is plotted at every time point. Standard deviations are represented as a shaded region around the means (n = 5). [file peerj-12-17179-s001.pdf]

Evaluation of a Cadaveric Wrist Motion Simulator using Marker-Based X-Ray Reconstruction of Moving Morphology: Supplementary Material

Table S1: Pre- and post- surgery wrist angles (°) in the starting positions of all planar motions. The X-Axis corresponds to pronation (+) and supination (-), the Y-Axis to flexion (+) and extension (-), the Z-Axis to ulnar (+) and radial (-) deviation. Angles are reported as the mean ± standard deviation. Statistically significant differences between anatomical and Motec wrists are indicated by a p-value < 0.05). N = 6.

		**Initial Wrist Positions (°)**					
		**Flexion**	**Extension**	**Radial Deviation**	**Ulnar Deviation**	**Radial Extension**	**Ulnar Flexion**
**X-Axis**	*Anatomical*	3.5 ± 3.8	3.0 ± 3.7	3.0 ± 5.3	3.3 ± 5.2	2.8 ± 4.0	3.6 ± 3.8
	Motec	3.1 ± 6.0	2.5 ± 5.7	2.8 ± 6.2	2.2 ± 6.5	2.0 ± 5.7	2.3 ± 7.2
	P-Value	0.873	0.812	0.951	0.623	0.734	0.627
**Y-Axis**	*Anatomical*	-10.9 ± 6.7	-9.8 ± 4.7	-11.9 ± 5.0	-11.3 ± 6.7	-11.4 ± 5.2	-11.1 ± 6.1
	Motec	-8.7 ± 9.1	-11.1 ± 7.3	-9.6 ± 7.7	-8.9 ± 8.4	-10.8 ± 7.4	-8.9 ± 8.6
	P-Value	0.245	0.323	0.143	0.083	0.583	0.201
**Z-Axis**	*Anatomical*	-0.7 ± 6.9	-0.4 ± 6.9	1.0 ± 7.5	0.4 ± 7.2	1.2 ± 7.3	-0.2 ± 7.2
	Motec	1.0 ± 7.9	1.6 ± 8.0	1.4 ± 8.0	1.7 ± 8.2	1.9 ± 7.4	1.7 ± 8.0
	P-Value	0.268	0.146	0.742	0.235	0.587	0.142

Table S2: Mean inter-specimen RMSEs (±standard deviation) of wrist angle (°) profiles in the X, Y and Z axes of the radial coordinate system. Highlighted cells indicate the axis with the maximum error during each motion (n = 6)

	**Mean Intra-Specimen RMSE (°)**					
	**Anatomical**			**Motec**		
	X-Axis	*Y-Axis*	*Z Axis*	*X-Axis*	*Y-Axis*	*Z Axis*
**Flexion**	3.97 ± 3.01	4.46 ± 2.11	4.67 ± 2.24	6.13 ± 3.12	5.45 ± 4.10	5.06 ± 3.62
**Extension**	2.95 ± 1.77	5.57 ± 2.47	4.67 ± 4.42	4.56 ± 3.25	10.7 ± 5.00	7.12 ± 6.05
**RD**	3.95 ± 2.61	5.12 ± 2.48	6.22 ± 3.44	6.00 ± 4.14	6.32 ± 5.72	7.28 ± 7.21
**UD**	3.98 ± 2.95	5.00 ± 4.64	5.17 ± 2.95	5.43 ± 2.11	8.60 ± 4.64	5.60 ± 4.32
**RE**	4.22 ± 2.57	7.85 ± 3.91	5.45 ± 4.65	8.16 ± 5.59	12.9 ± 6.42	7.11 ± 5.00
**UF**	3.18 ± 2.36	5.61 ± 2.52	6.05 ± 2.39	6.29 ± 3.49	5.79 ± 3.57	5.54 ± 3.51
**Circumduction**	4.65 ± 2.25	7.07 ± 2.19	5.64 ± 2.54	7.87 ± 3.50	11.5 ± 5.05	7.32 ± 4.24

Table S3: Mean inter-specimen RMSEs (±standard deviation) of the force (N) profiles of ECU, ECRB, ECRL, FCR and FCU. Highlighted cells indicate the tendon with the maximum error during each motion (n = 6)

	Mean Intra-Specimen RMSE (N)										
	Anatomical						Motec				
	ECU	*ECRB*	*ECRL*	*FCR*	*FCU*		*ECU*	*ECRB*	*ECRL*	*FCR*	*FCU*
**Flexion**	0.36 ± 0.21	0.57± 0.27	0.23± 0.12	2.84± 0.75	3.49± 1.45		0.42± 0.24	1.57± 0.78	0.52± 0.39	1.76± 1.25	2.54± 0.77
**Extension**	4.85± 2.31	4.52± 2.79	3.39± 1.52	0.31± 0.16	0.33± 0.17		5.39± 4.19	4.16± 2.26	6.25± 3.78	0.51± 0.27	0.57± 0.21
**RD**	1.16± 0.36	3.09± 2.40	5.94± 2.58	4.10± 1.84	0.48± 0.22		1.36± 0.44	3.61± 1.55	8.45± 4.26	5.22± 4.17	0.60± 0.38
**UD**	3.97± 2.93	0.37± 0.27	0.44± 0.18	0.21± 0.09	1.82± 1.14		7.21± 3.59	0.47± 0.21	0.54± 0.24	0.23± 0.07	1.44± 1.24
**RE**	3.05± 1.79	5.74± 1.70	6.23± 4.22	9.29± 3.61	0.37± 0.14		3.95± 2.36	4.40± 1.73	9.14± 7.54	5.30± 3.23	0.83± 0.43
**UF**	1.50± 0.50	2.09± 1.02	0.24± 0.13	0.57± 0.41	3.05± 1.23		1.00± 0.43	1.07± 0.63	0.47± 0.34	0.68± 0.58	3.13± 0.99
**Circumduction**	5.49± 2.09	3.30± 1.60	5.02± 3.43	3.48± 2.28	3.98± 1.48		5.90± 1.89	4.61± 2.51	8.41± 3.33	1.84± 1.29	3.79± 1.55

Table S4: Mean inter-specimen RMSEs (±standard deviation) of the actuator displacement (mm) profiles of ECU, ECRB, ECRL, FCR and FCU tendons. Highlighted cells indicate the tendon with the maximum error during each motion (n = 6)

	Mean Intra-Specimen RMSE (mm)				
	ECU	*ECRB*	*ECRL*	*FCR*	*FCU*
**Flexion**	0.56 ± 0.18	0.56 ± 0.18	0.57 ± 0.17	1.59 ± 0.31	1.07 ± 0.57
**Extension**	0.61 ± 0.40	0.69 ± 0.08	0.64 ± 0.37	0.62 ± 0.05	0.62 ± 0.05
**RD**	0.55 ± 0.27	0.62 ± 0.56	0.97 ± 0.62	0.83 ± 0.55	0.55 ± 0.27
**UD**	0.28 ± 0.26	0.11 ± 0.10	0.11 ± 0.10	0.11 ± 0.10	1.92 ± 0.79
**RE**	0.51 ± 0.46	1.00 ± 0.99	1.23 ± 1.14	4.20 ± 2.04	0.49 ± 0.49
**UF**	1.64 ± 0.61	1.21 ± 1.15	0.48 ± 0.44	1.80 ± 1.52	0.79 ± 0.64
**Circumduction**	1.35 ± 1.08	1.05 ± 0.84	1.60 ± 1.41	2.81 ± 1.00	2.69 ± 0.54

The following graphs show the wrist angles, tendon forces and actuator displacements during the an average trial from every motion and specimen before (A, C & E) and after (B, D & F) a total wrist replacement. This excludes the radial extension trial of specimen 1, which is included as a representative trial in the main text. For every figure **A&B** correspond to angles (°) of the third metacarpal with respect to the radius, represented by rotations about the X (pronation/supination), Y (flexion/extension) and Z (radioulnar deviation) axes of the radial coordinate system. **C&D** correspond to forces (N) applied to the ECU, ECRB, ECRL, FCU and FCR tendons over the duration of the motion trial. **E&F** correspond to displacements (mm) of the five linear actuators connected to the tendons over the duration of the motion trial. The mean (s.d.) angle/force/displacement is plotted at every time point. Standard deviations are represented as a shaded region around the means (n = 5).

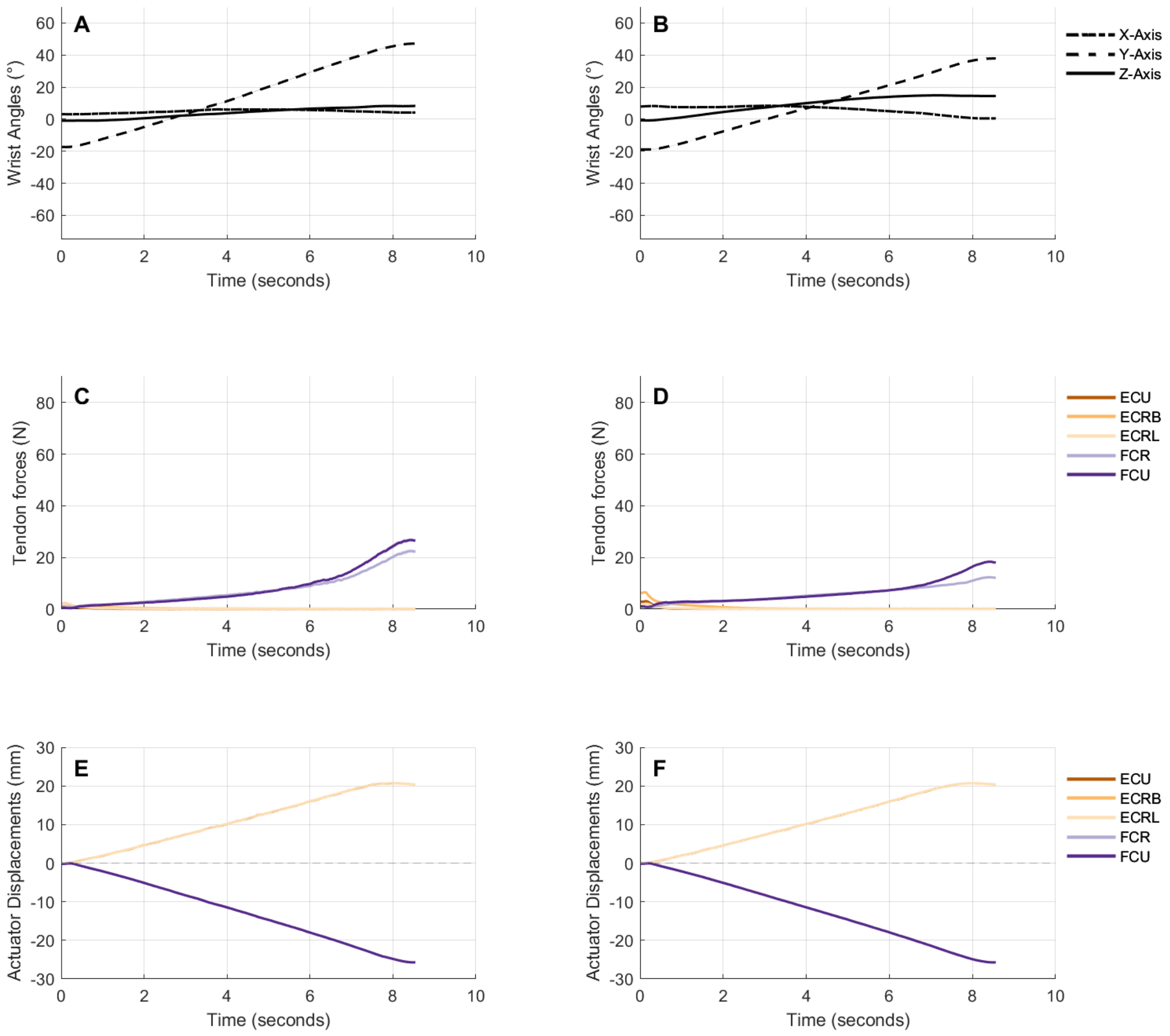

Figure S1: Average flexion trial of specimen 1.

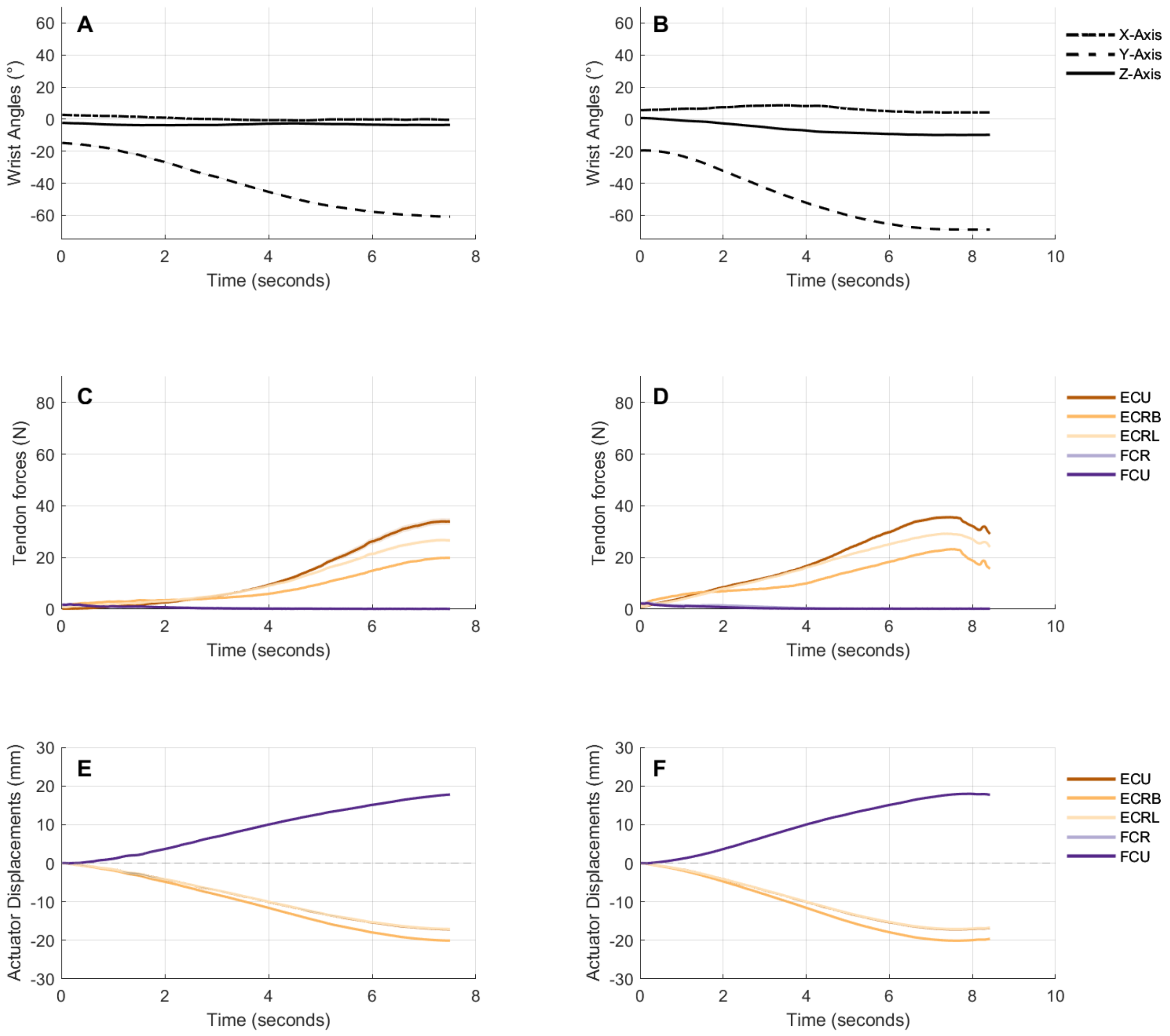

Figure S2: Average extension trial of specimen 1.

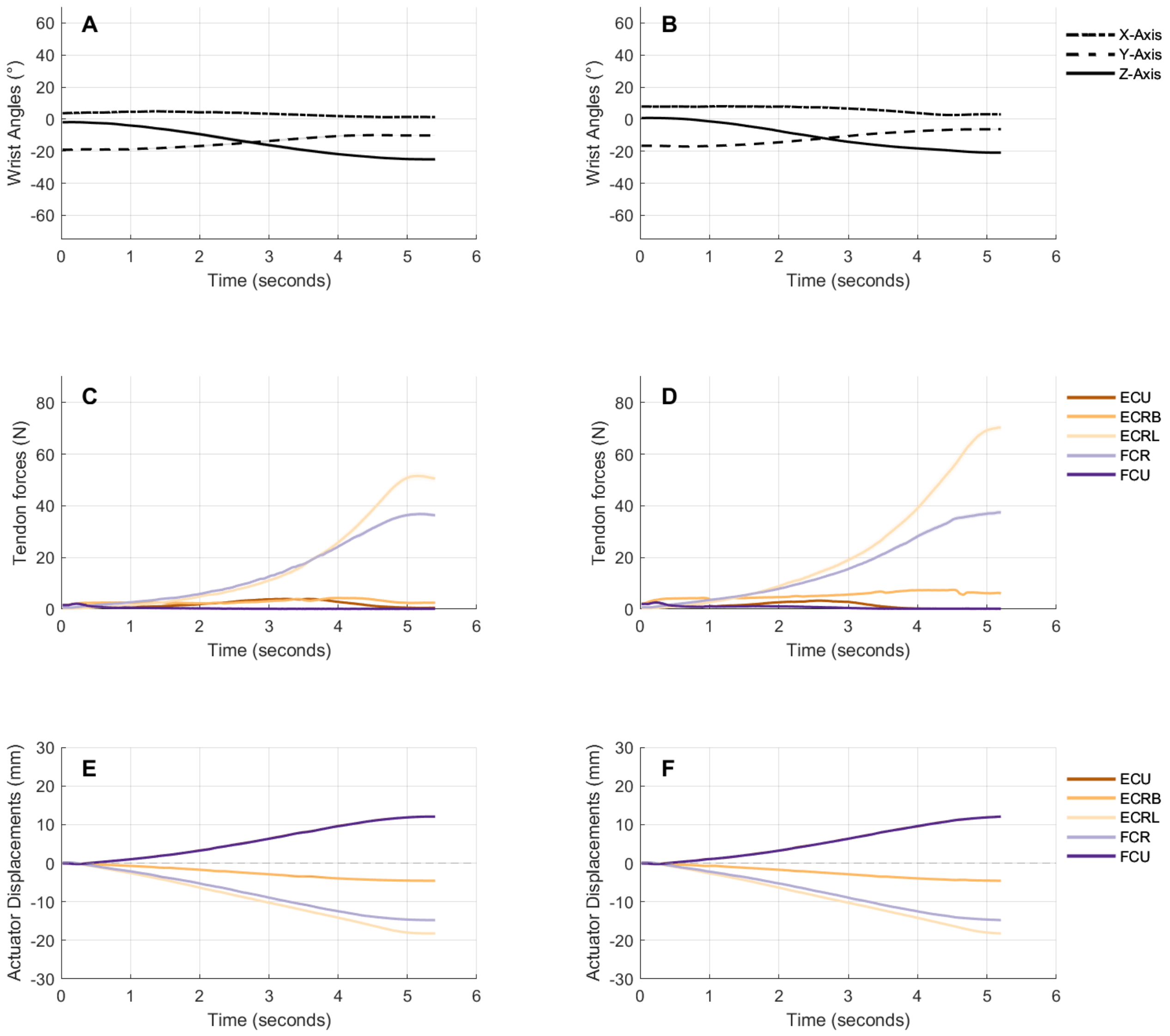

Figure S3: Average radial deviation trial of specimen 1.

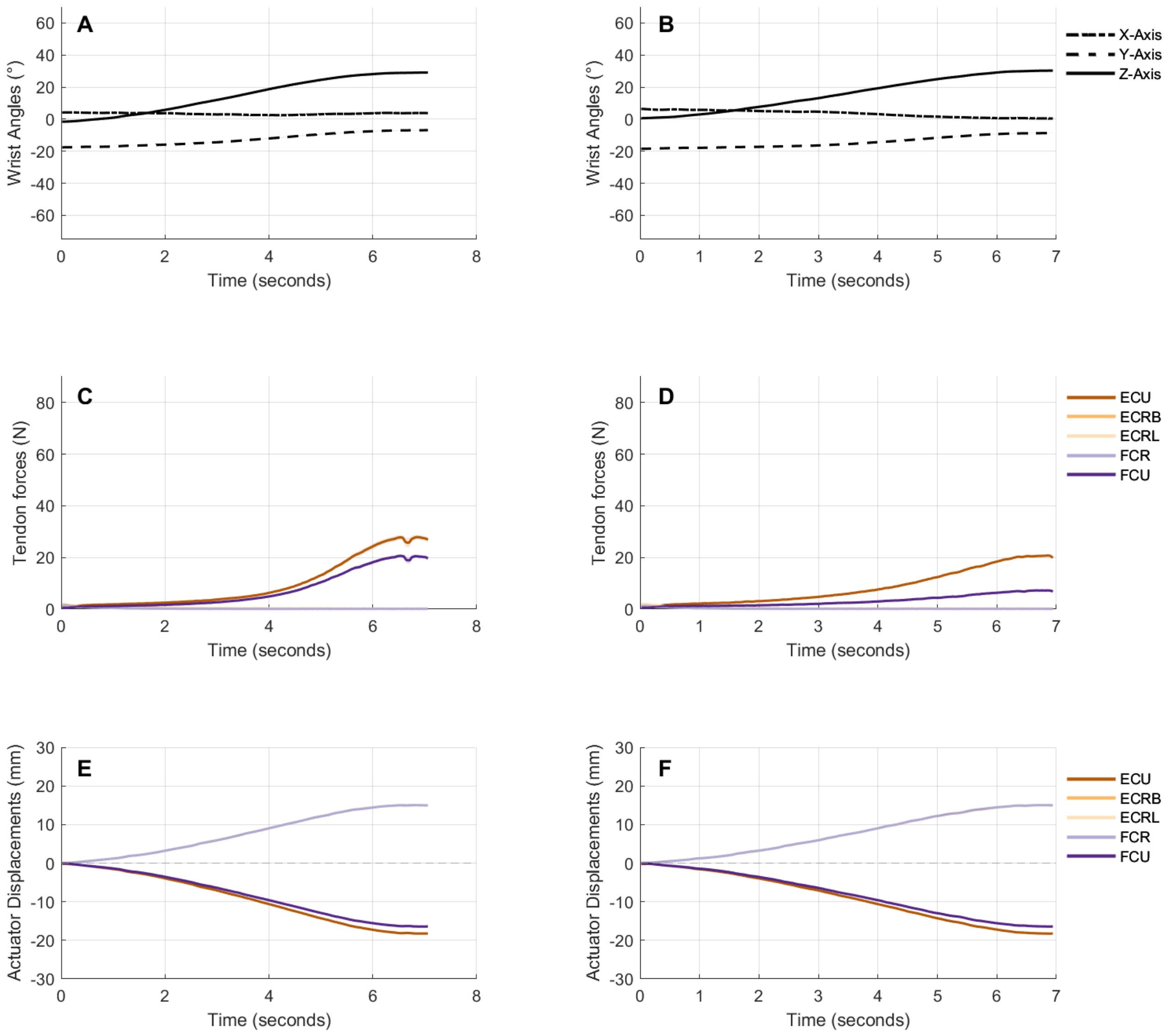

Figure S4: Average ulnar deviation trial of specimen 1.

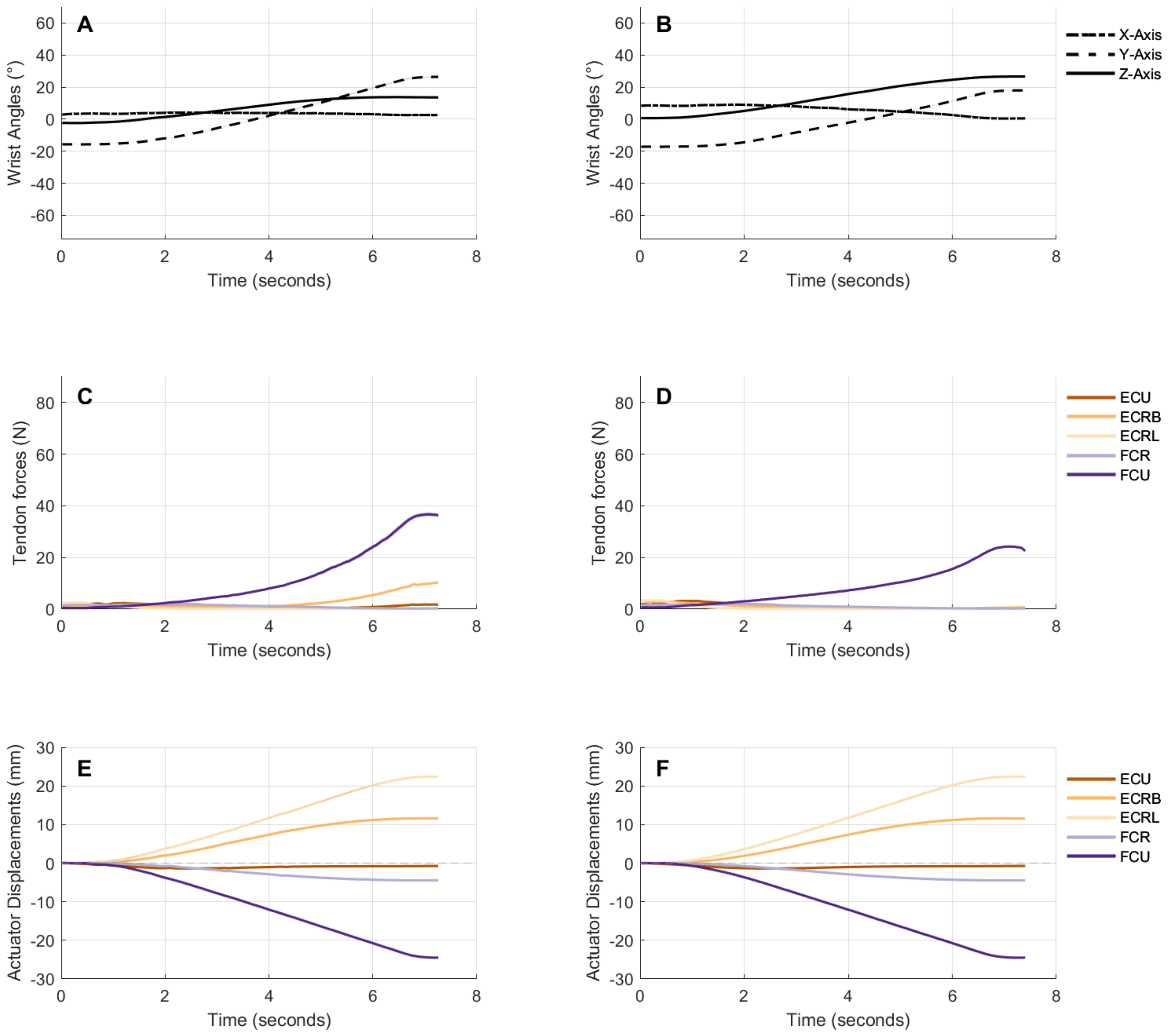

Figure S5: Average ulnar flexion trial of specimen 1.

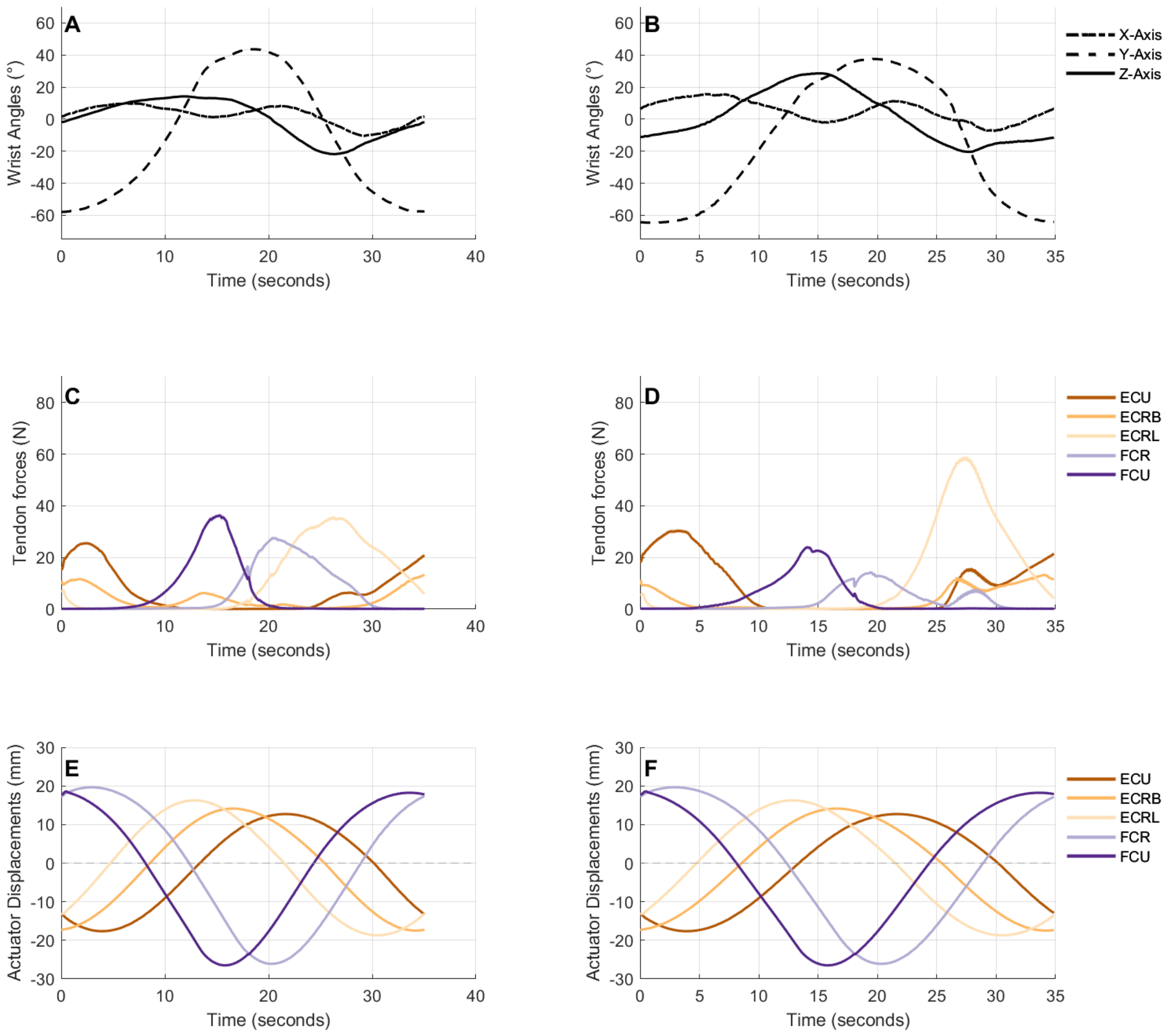

Figure S6: Average circumduction trial of specimen 1.

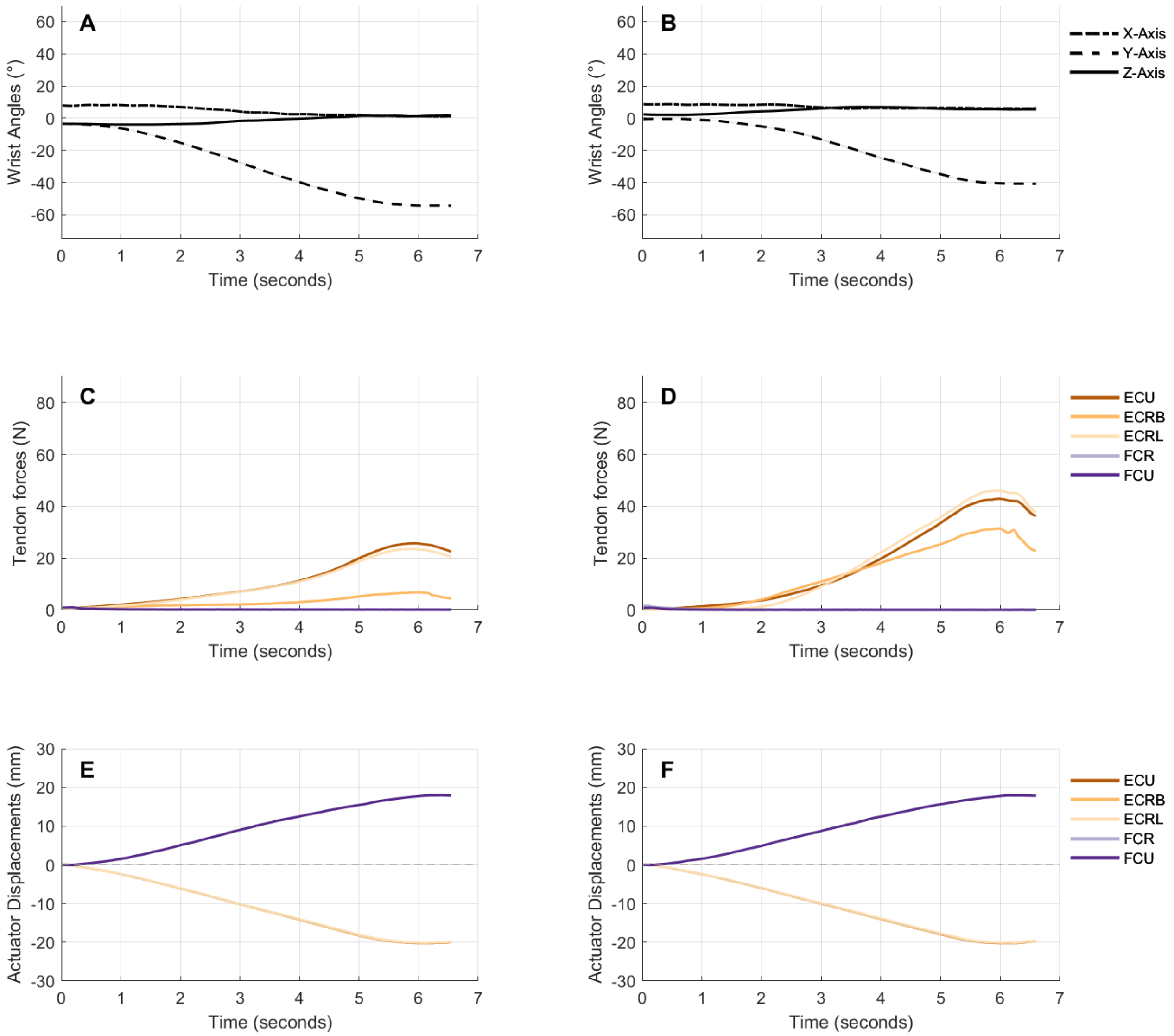

Figure S7: Average extension trial of specimen 2.

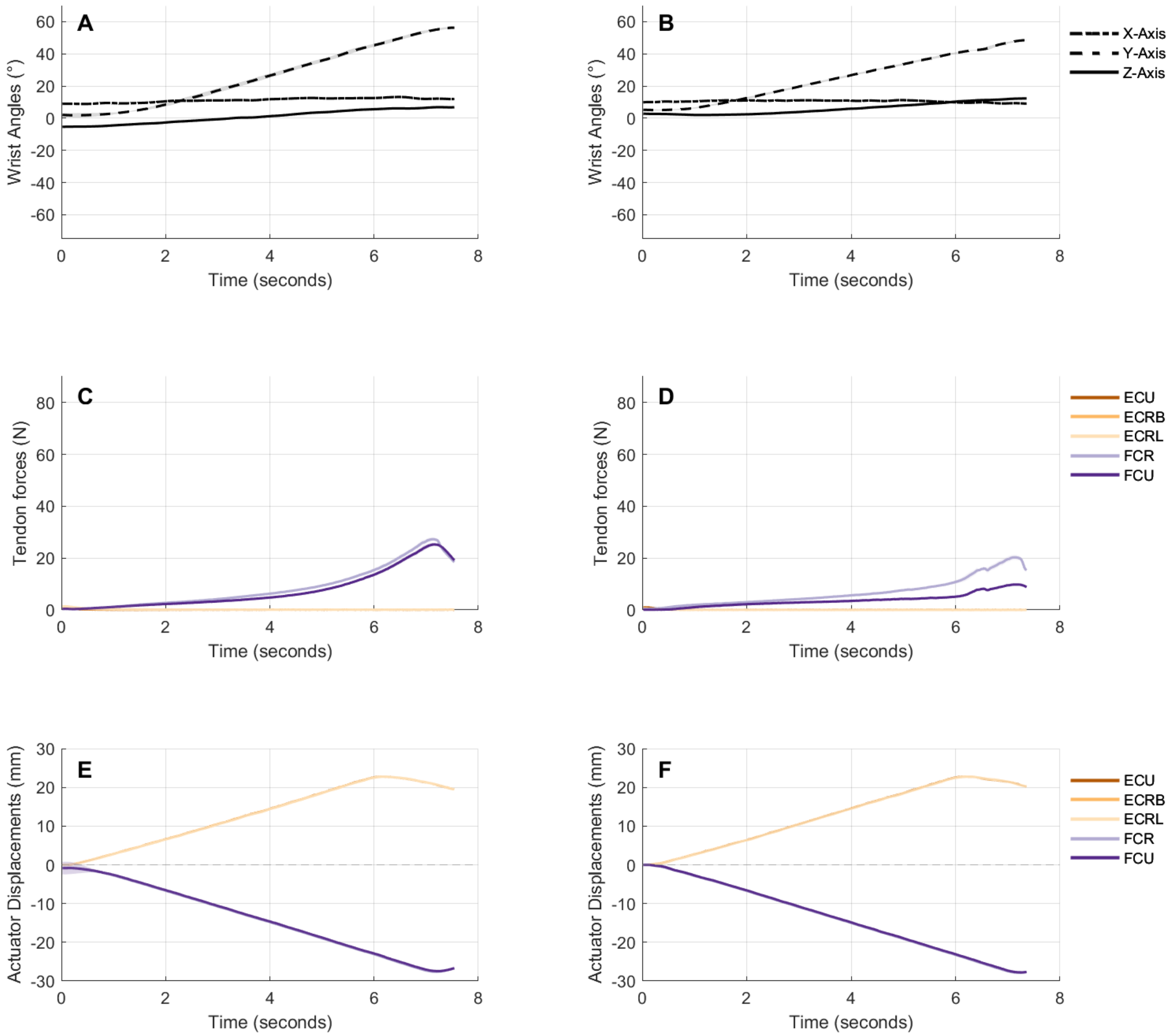

Figure S8: Average flexion trial of specimen 2.

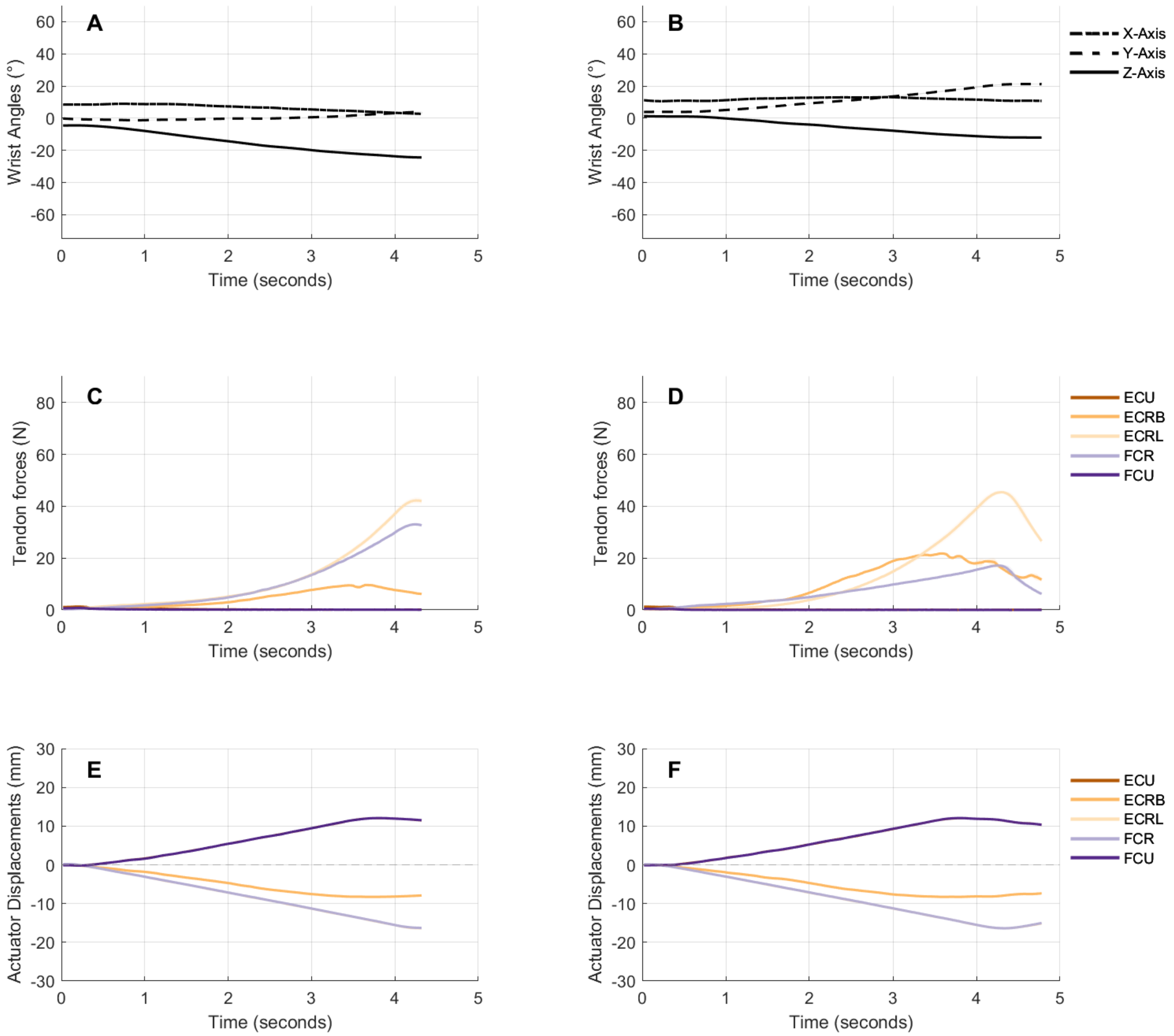

Figure S9: Average radial deviation trial of specimen 2.

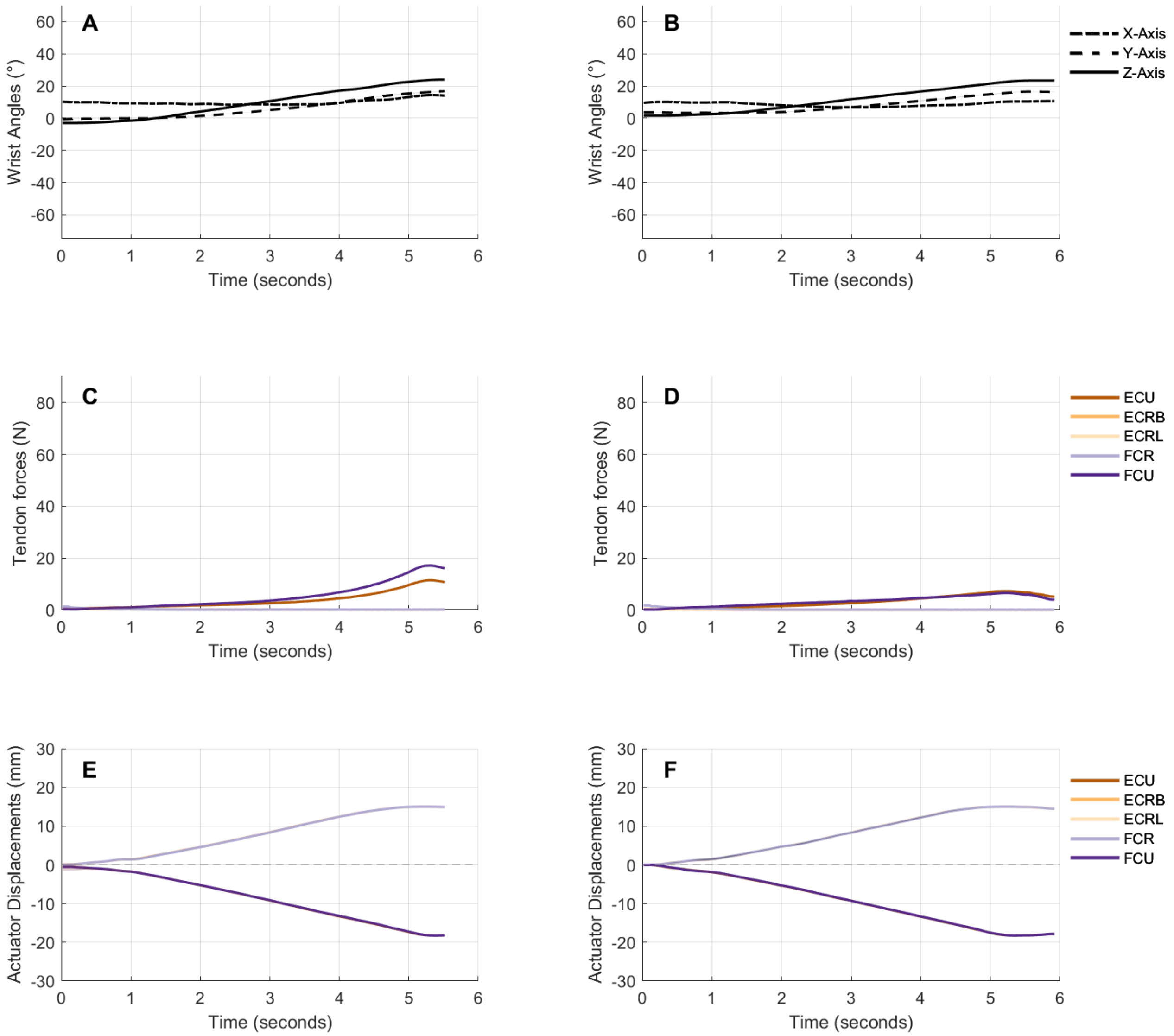

Figure S10: Average ulnar deviation trial of specimen 2.

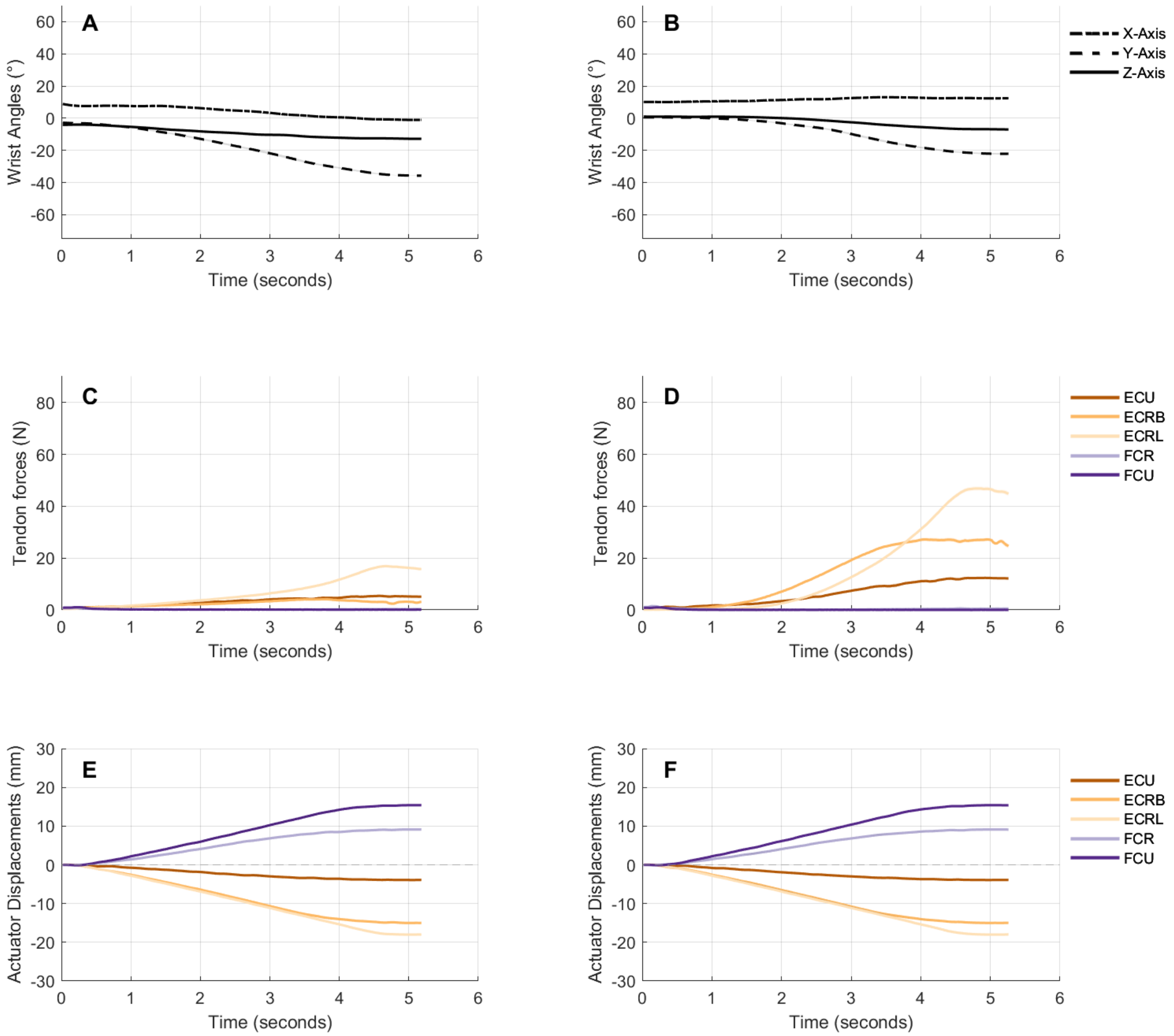

Figure S11: Average radial extension trial of specimen 2.

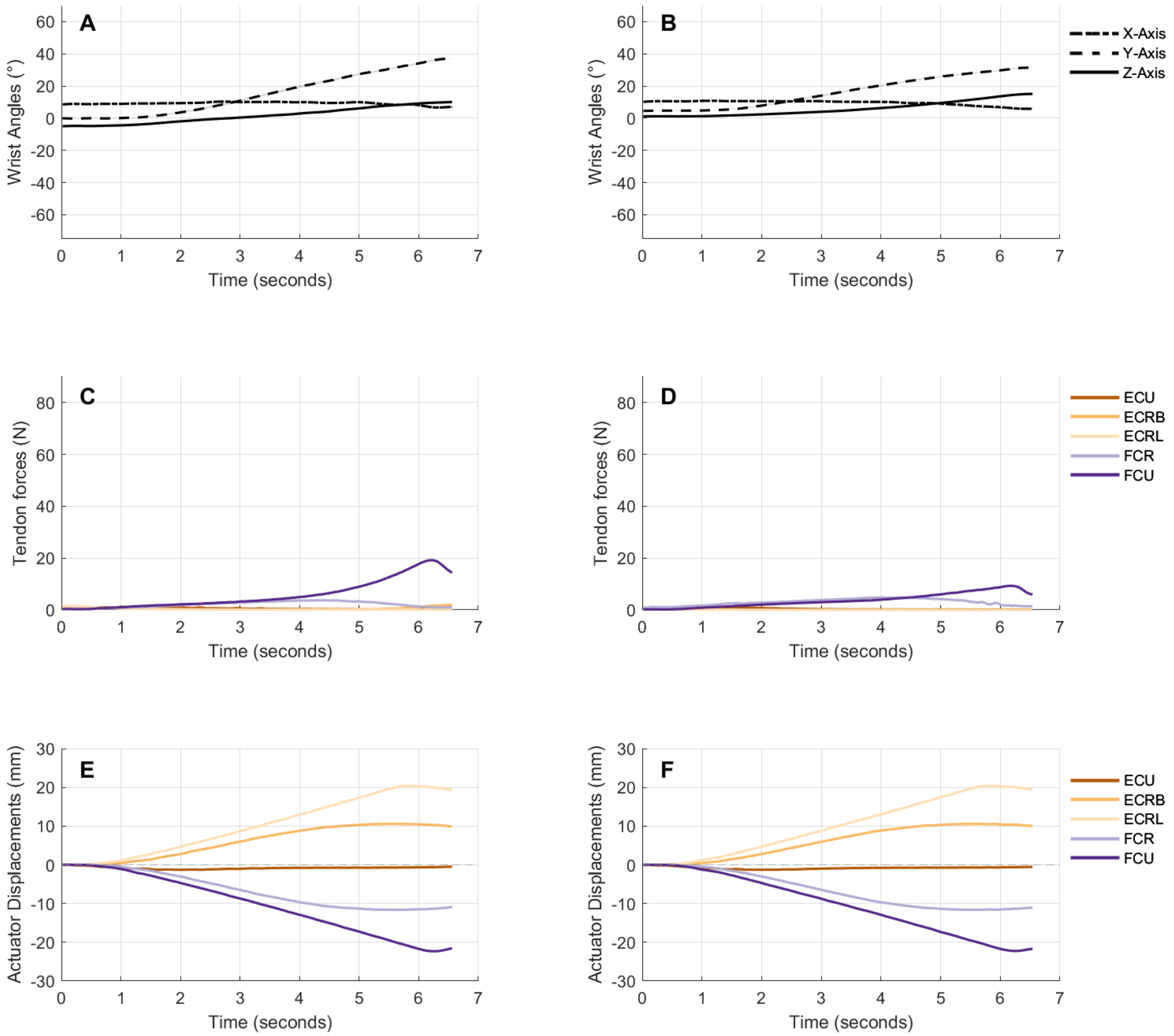

Figure S12: Average ulnar flexion trial of specimen 2.

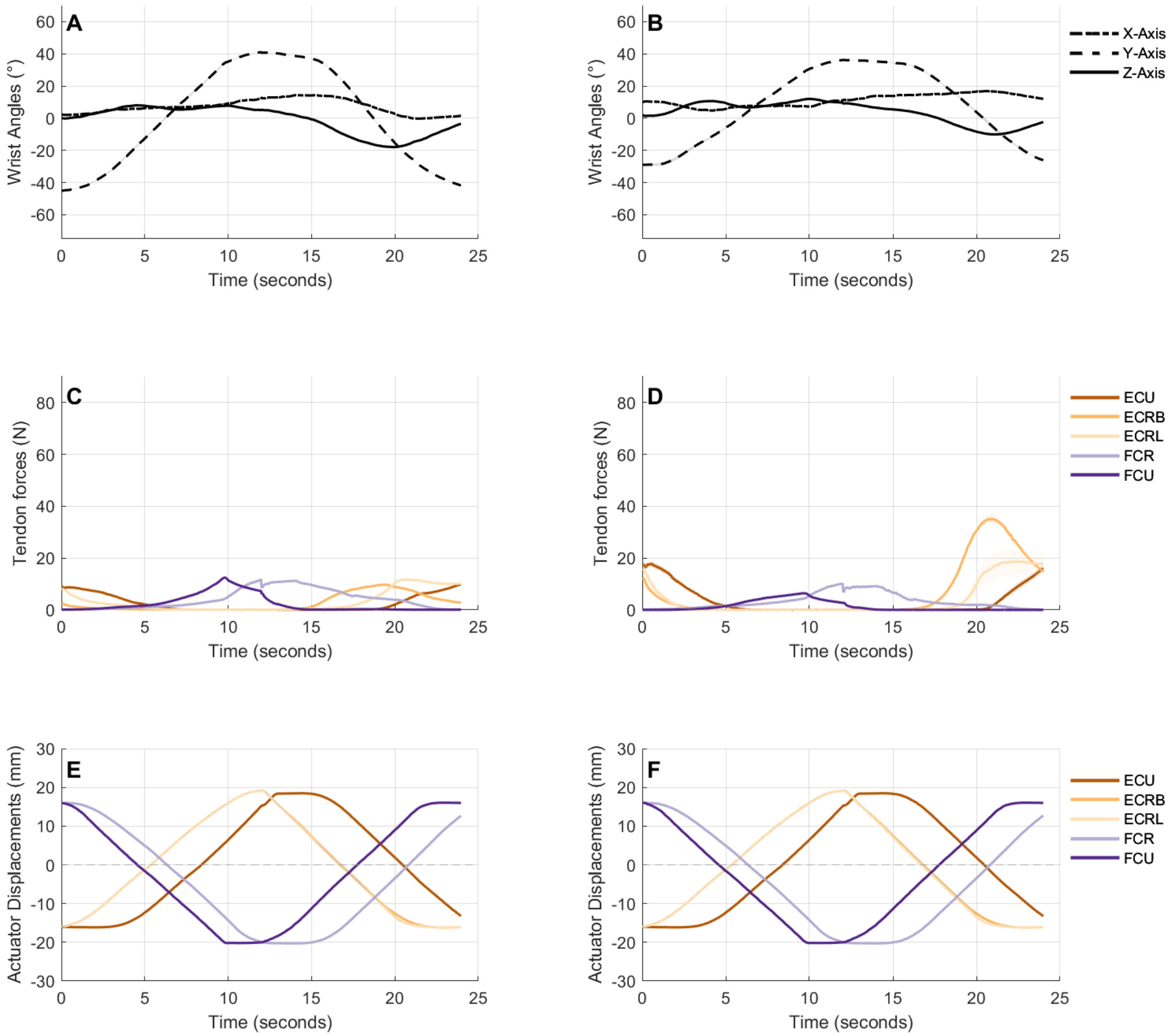

Figure S13: Average circumduction trial of specimen 2.

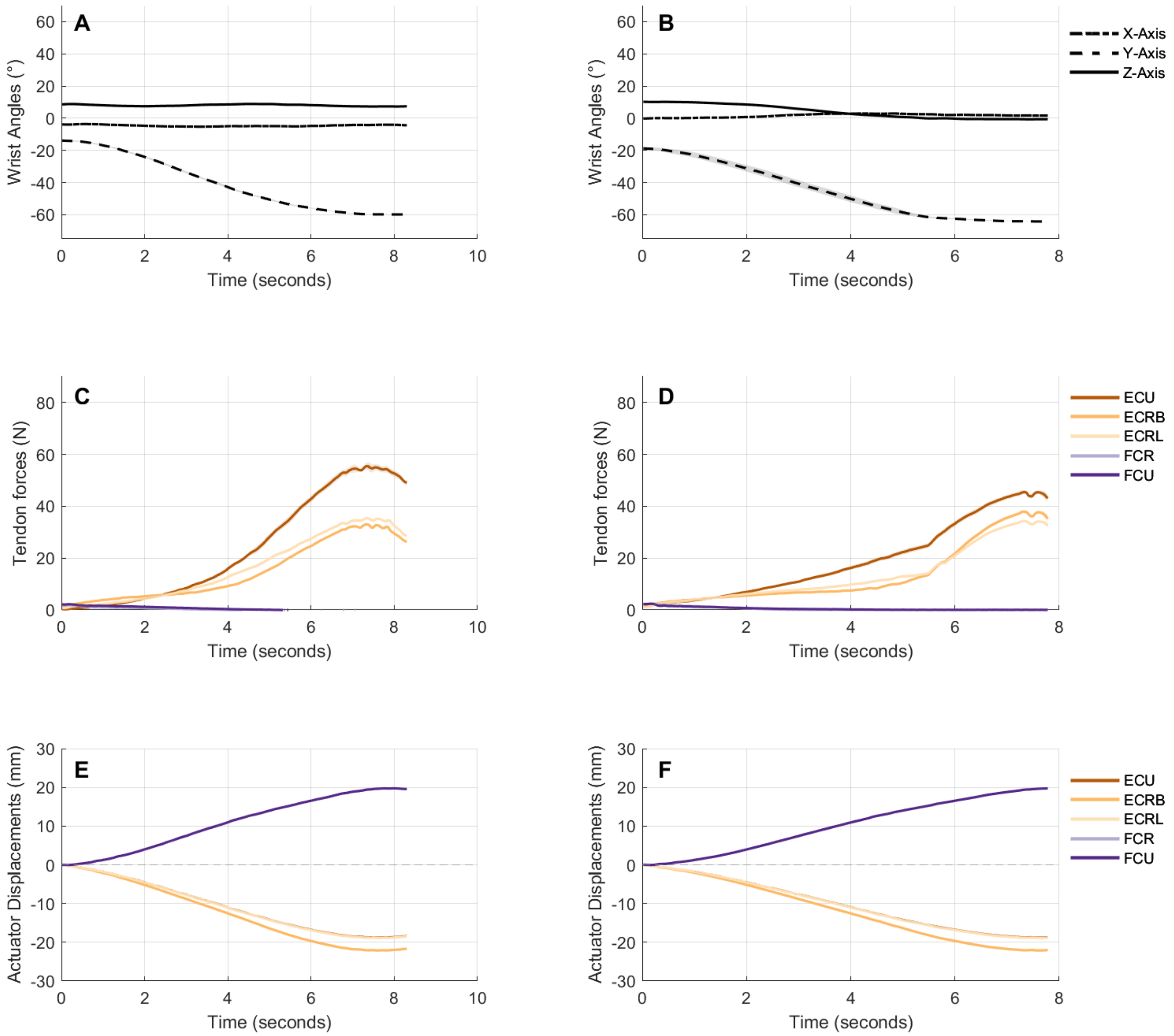

Figure S14: Average extension trial of specimen 3.

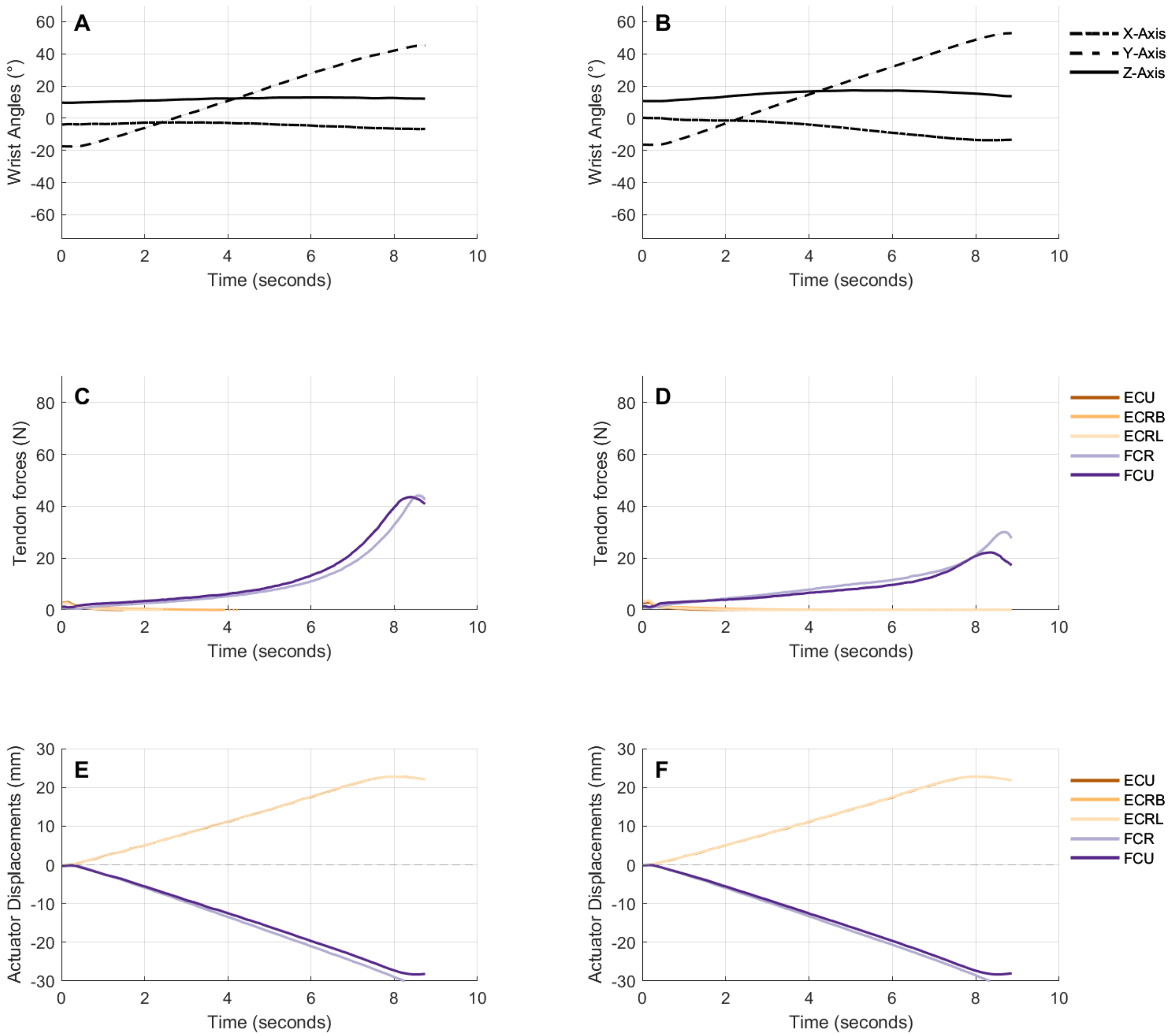

Figure S15: Average flexion trial of specimen 3.

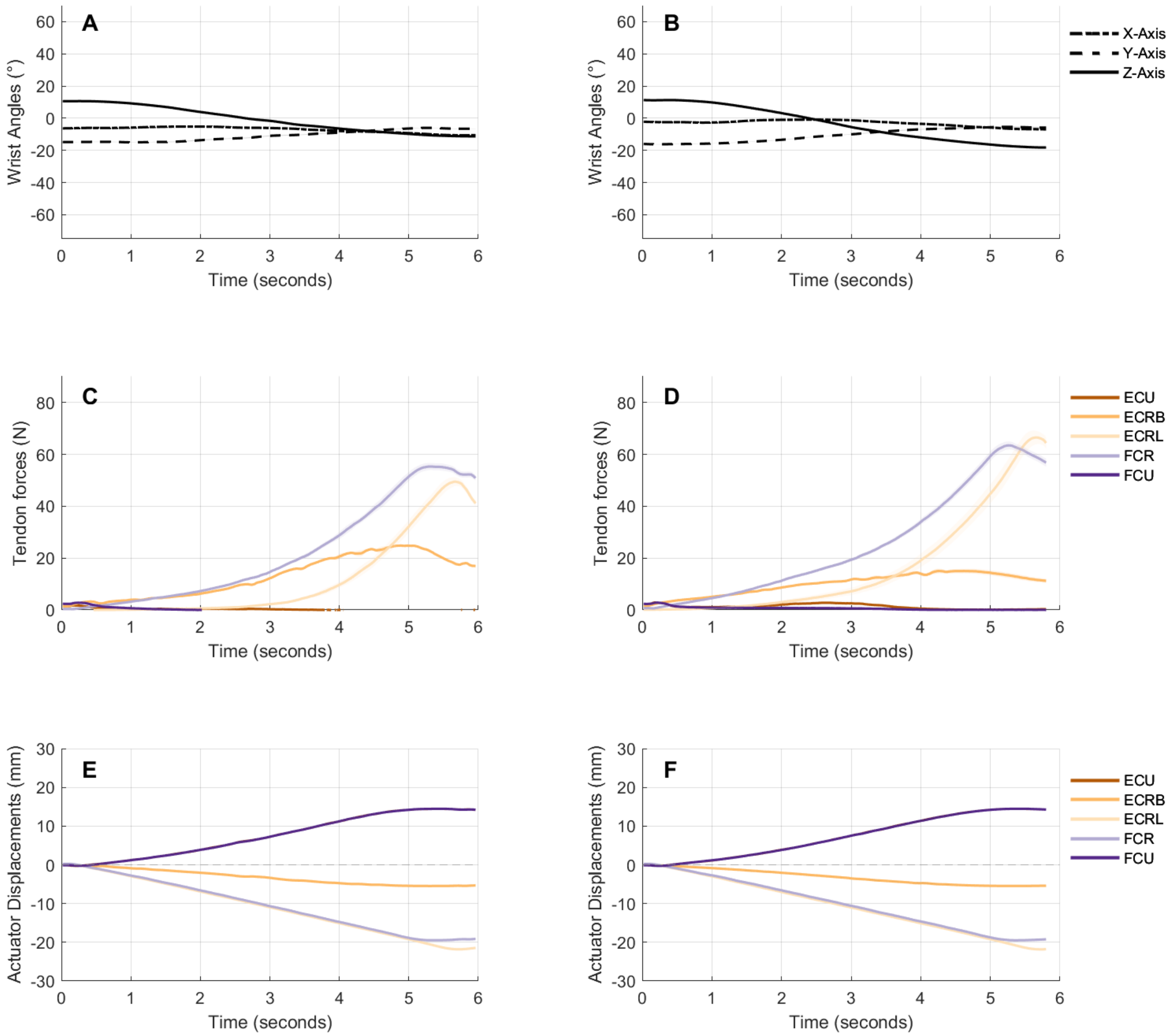

Figure S16: Average radial deviation trial of specimen 3.

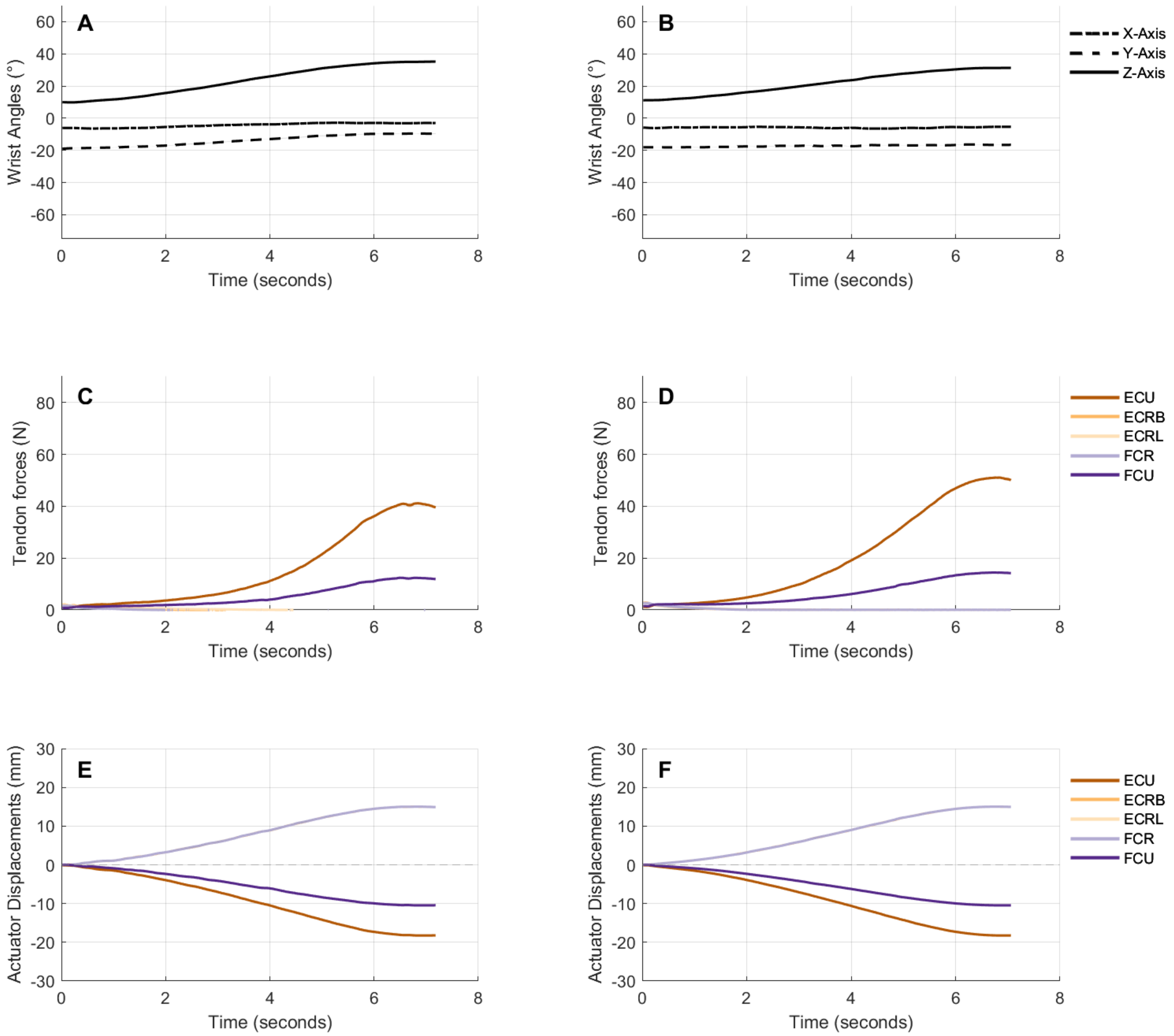

Figure S17: Average ulnar deviation trial of specimen 3.

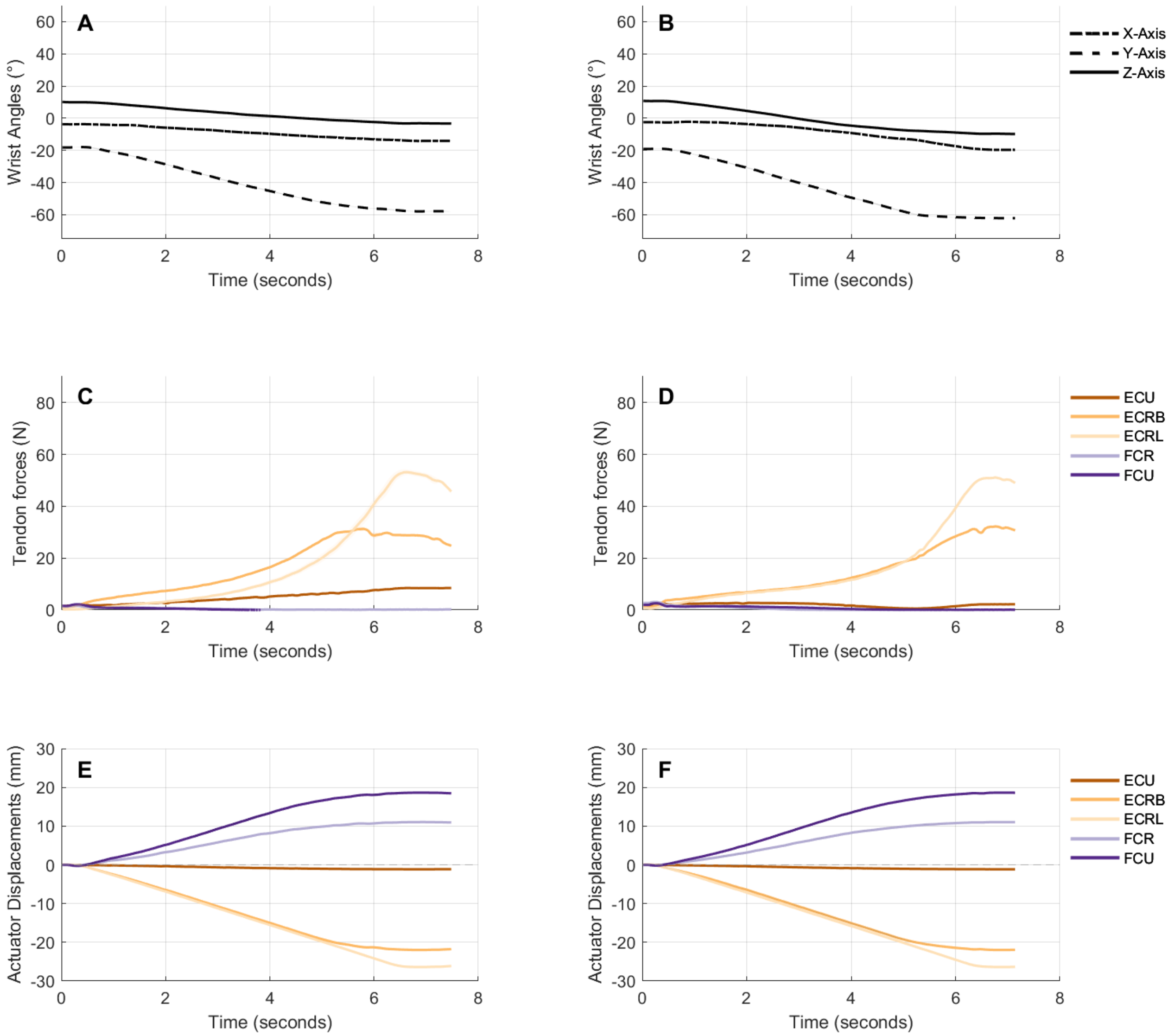

Figure S18: Average radial extension trial of specimen 3.

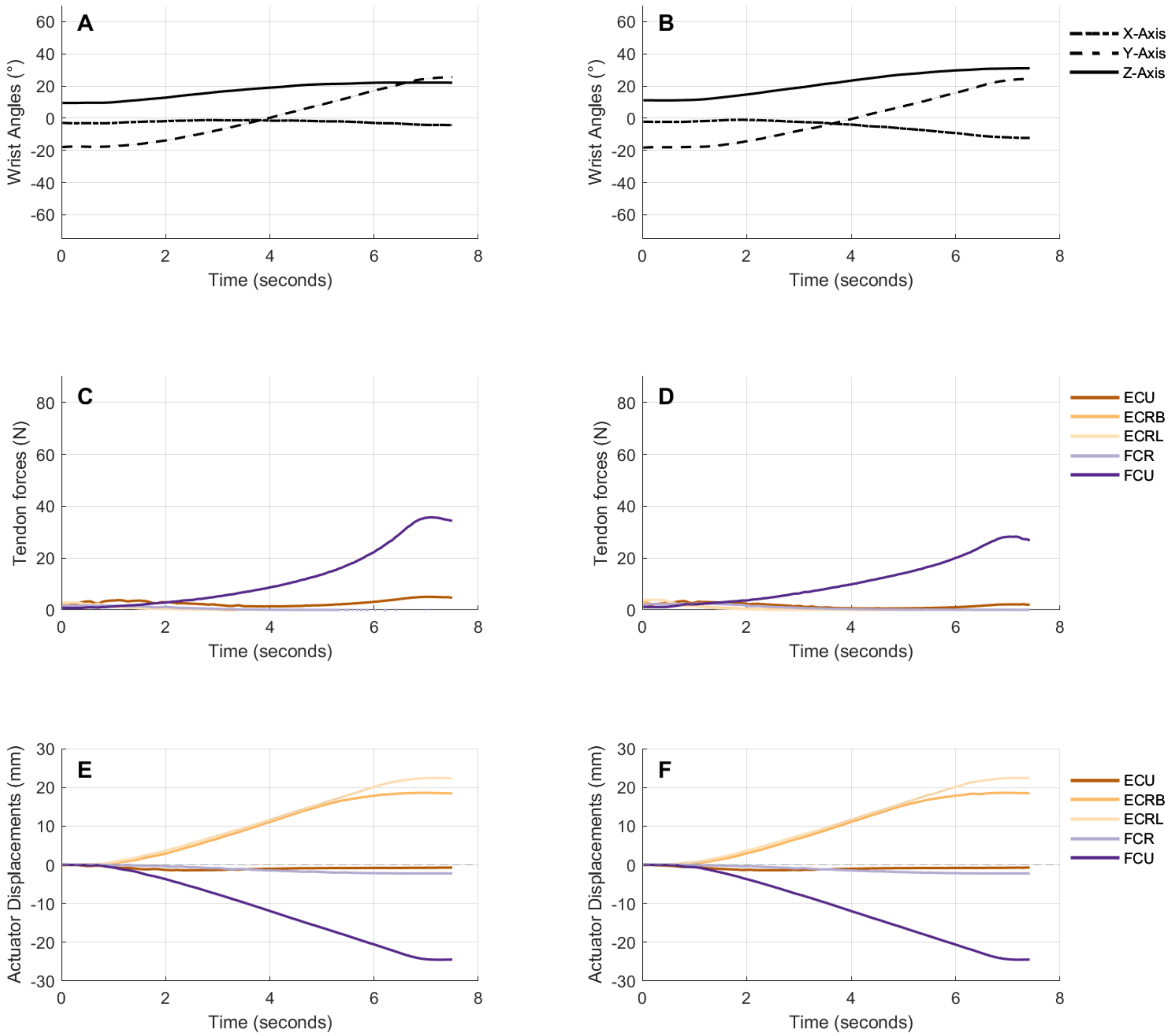

Figure S19: Average ulnar flexion trial of specimen 3.

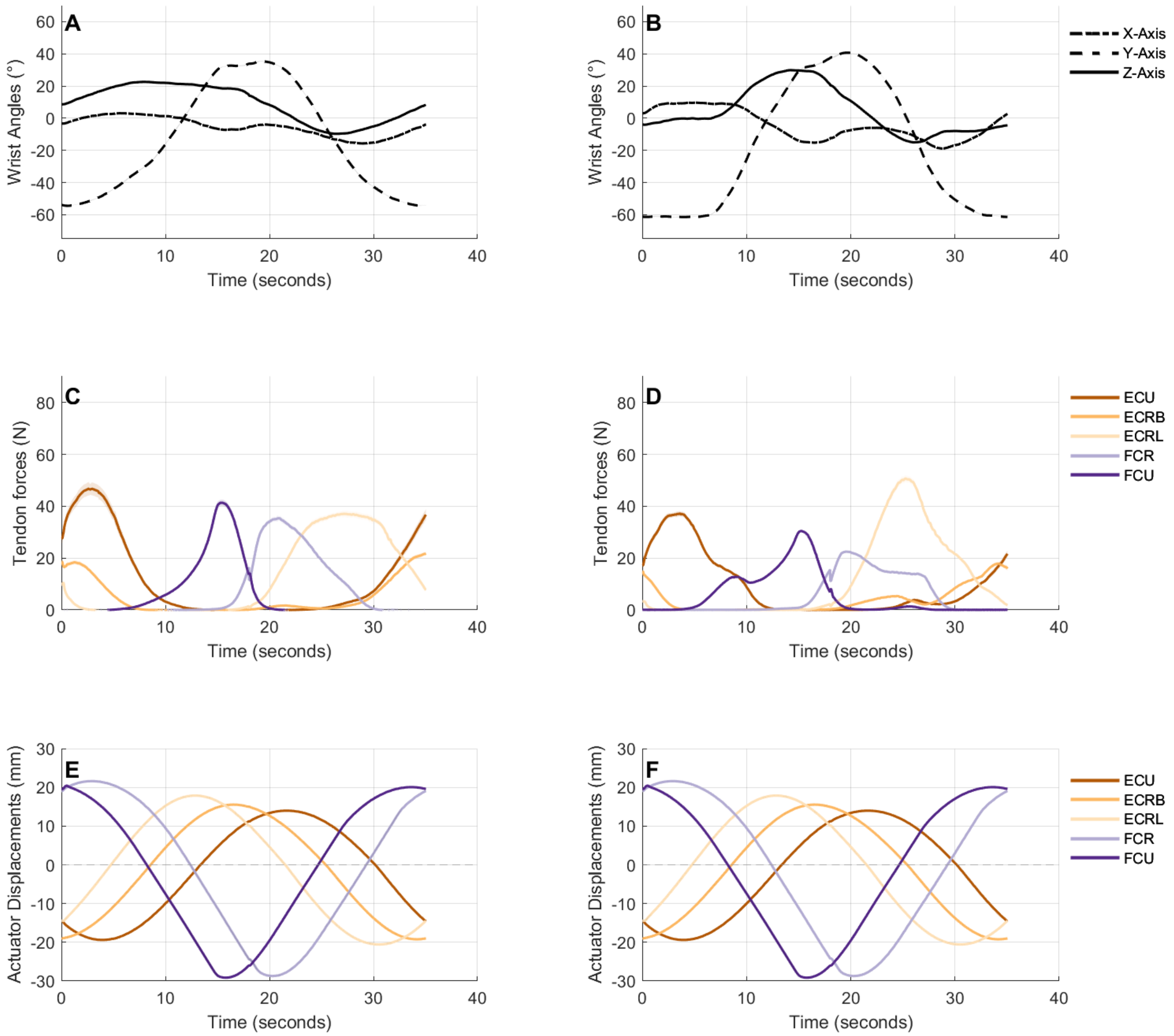

Figure S20: Average circumduction trial of specimen 3.

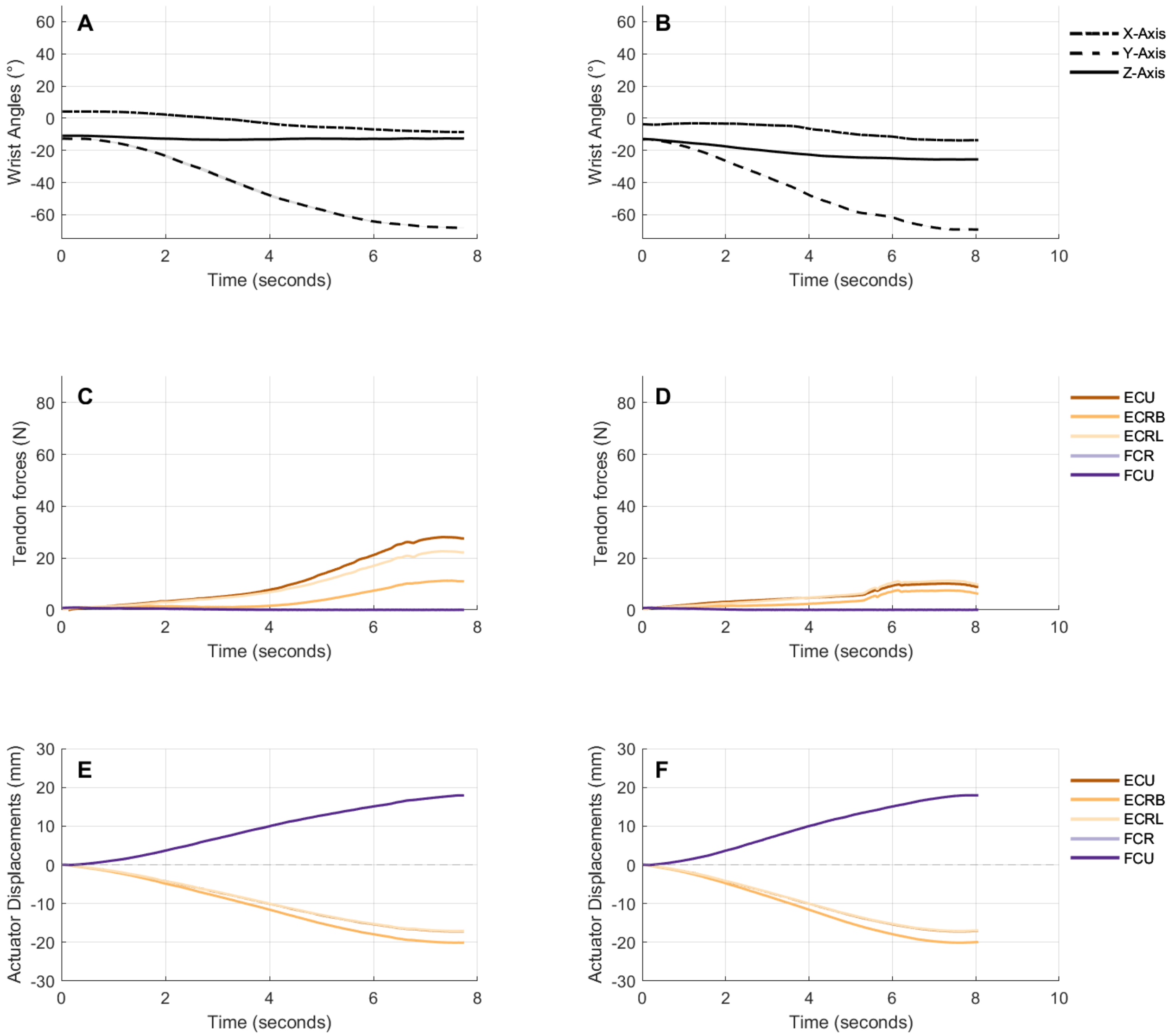

Figure S21: Average extension trial of specimen 4.

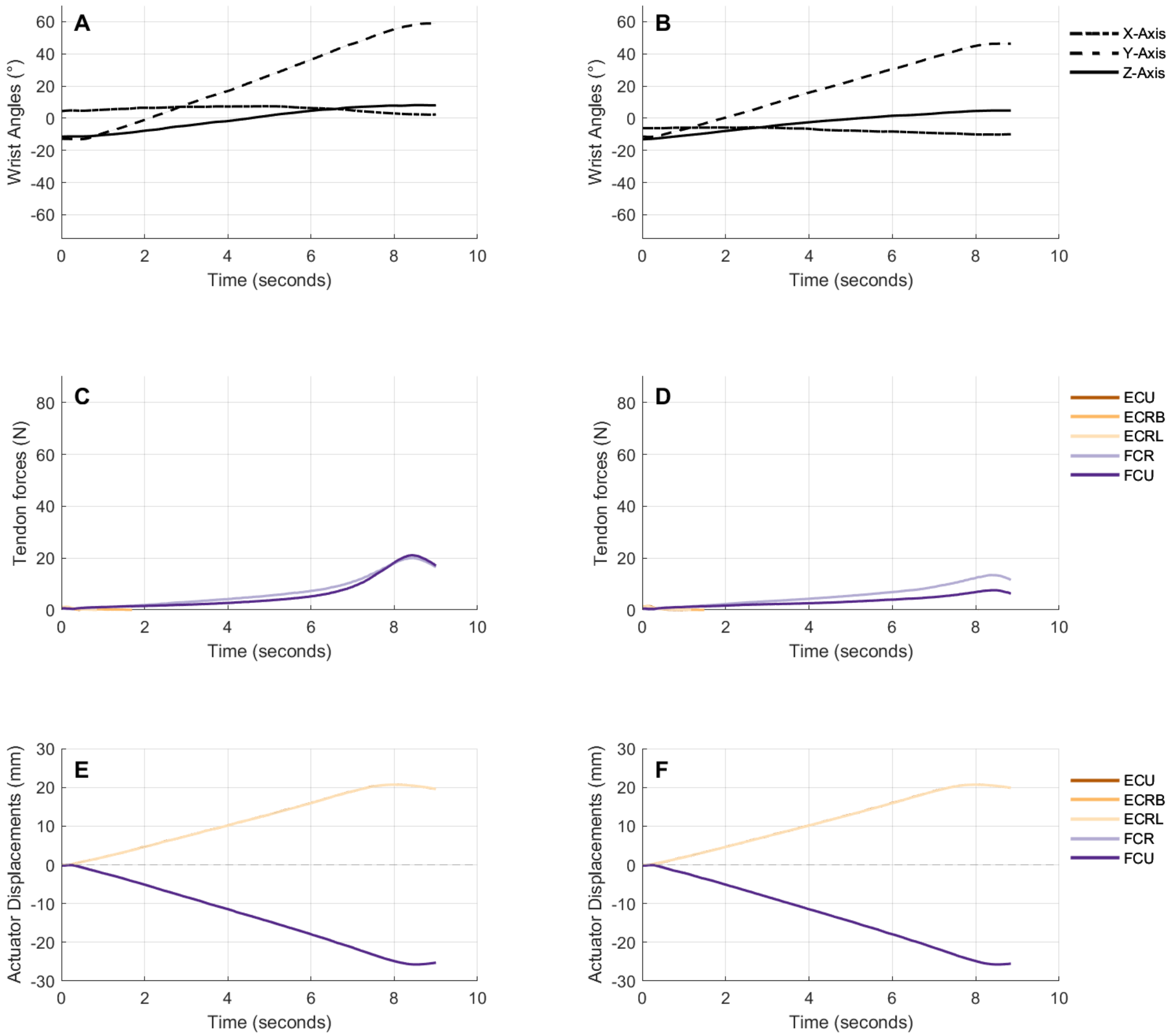

Figure S22: Average flexion trial of specimen 4.

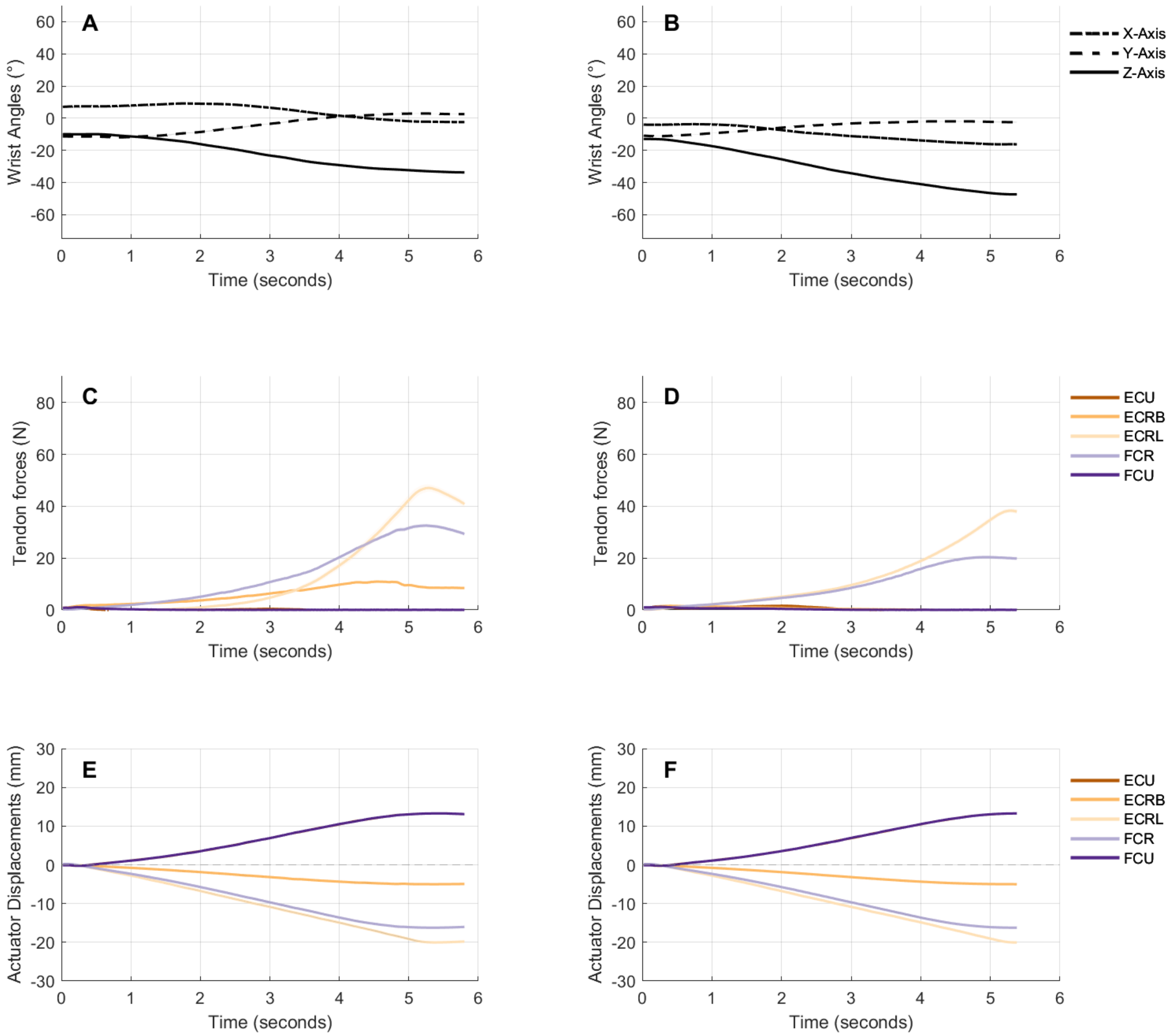

Figure S23: Average radial deviation trial of specimen 4.

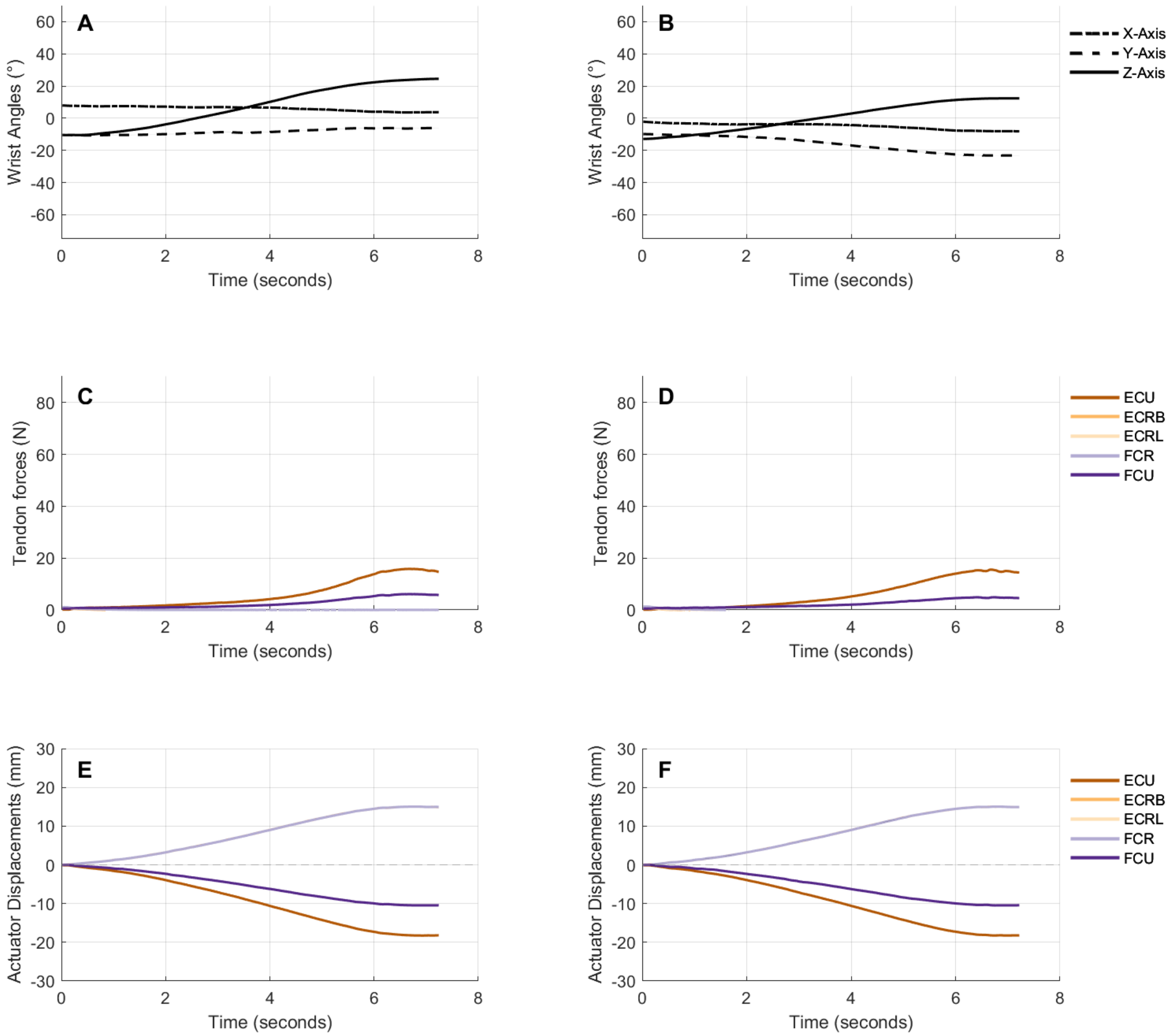

Figure S24: Average ulnar deviation trial of specimen 4.

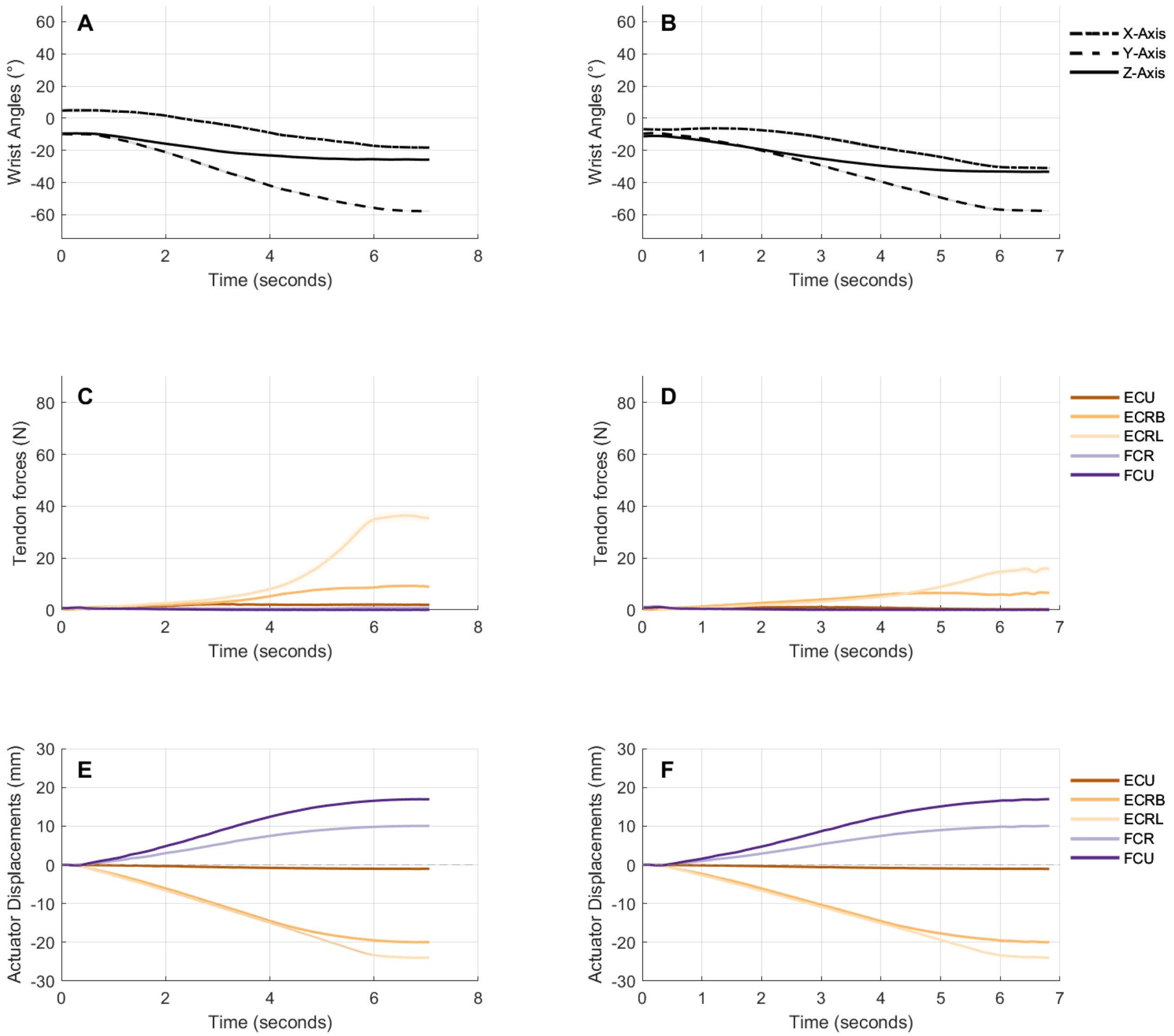

Figure S25: Average radial extension trial of specimen 4.

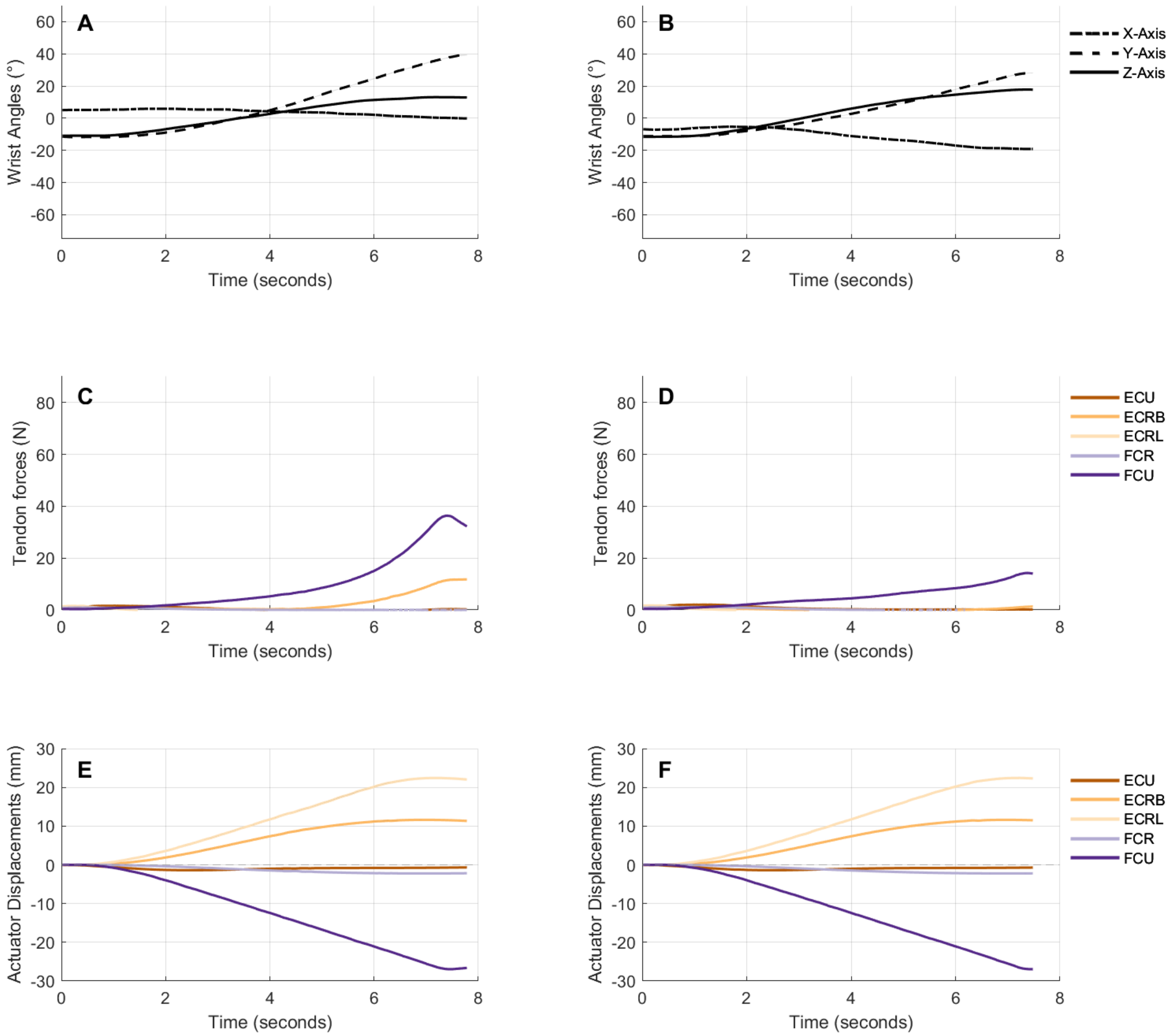

Figure S26: Average ulnar flexion trial of specimen 4.

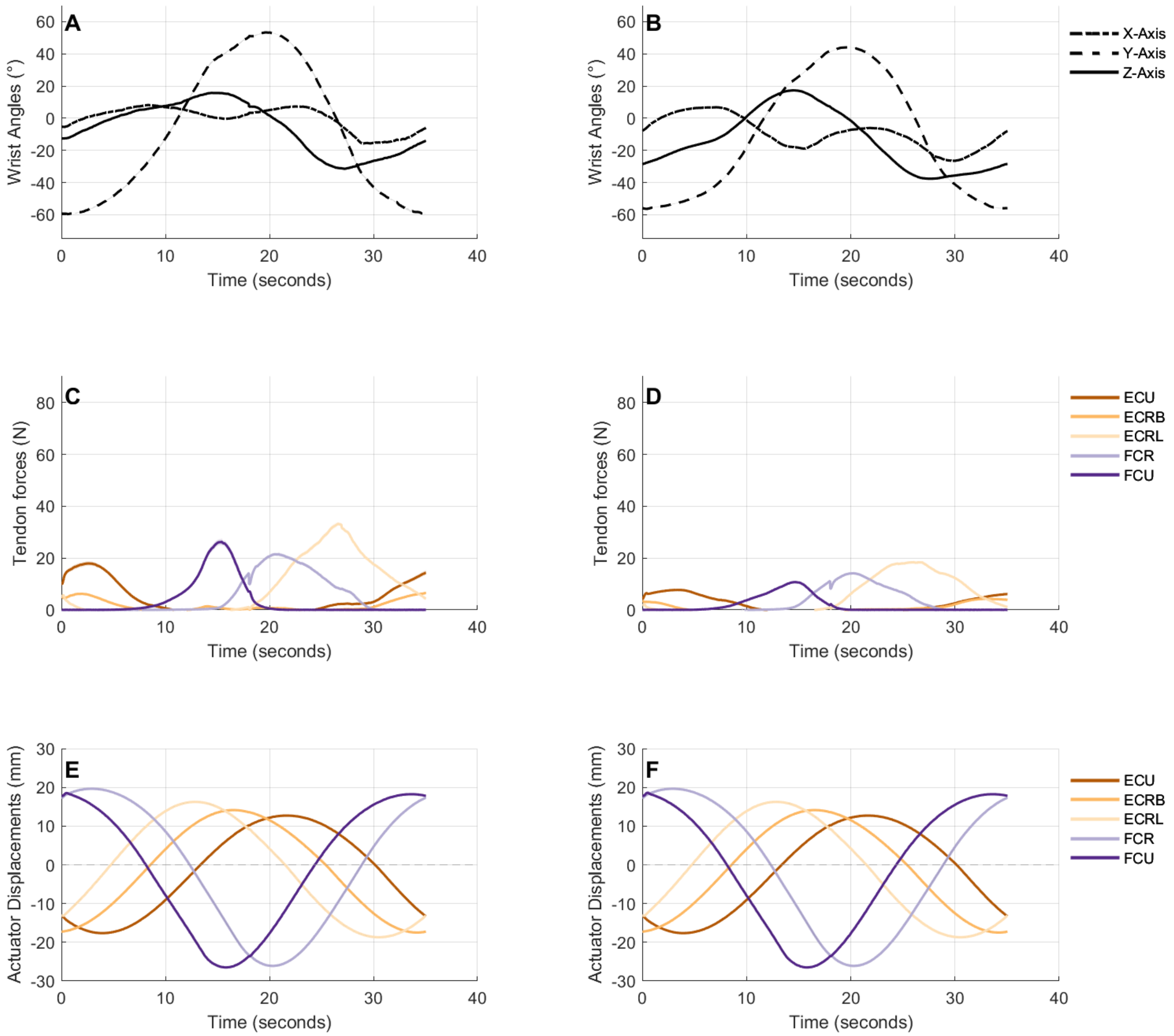

Figure S27: Average circumduction trial of specimen 4.

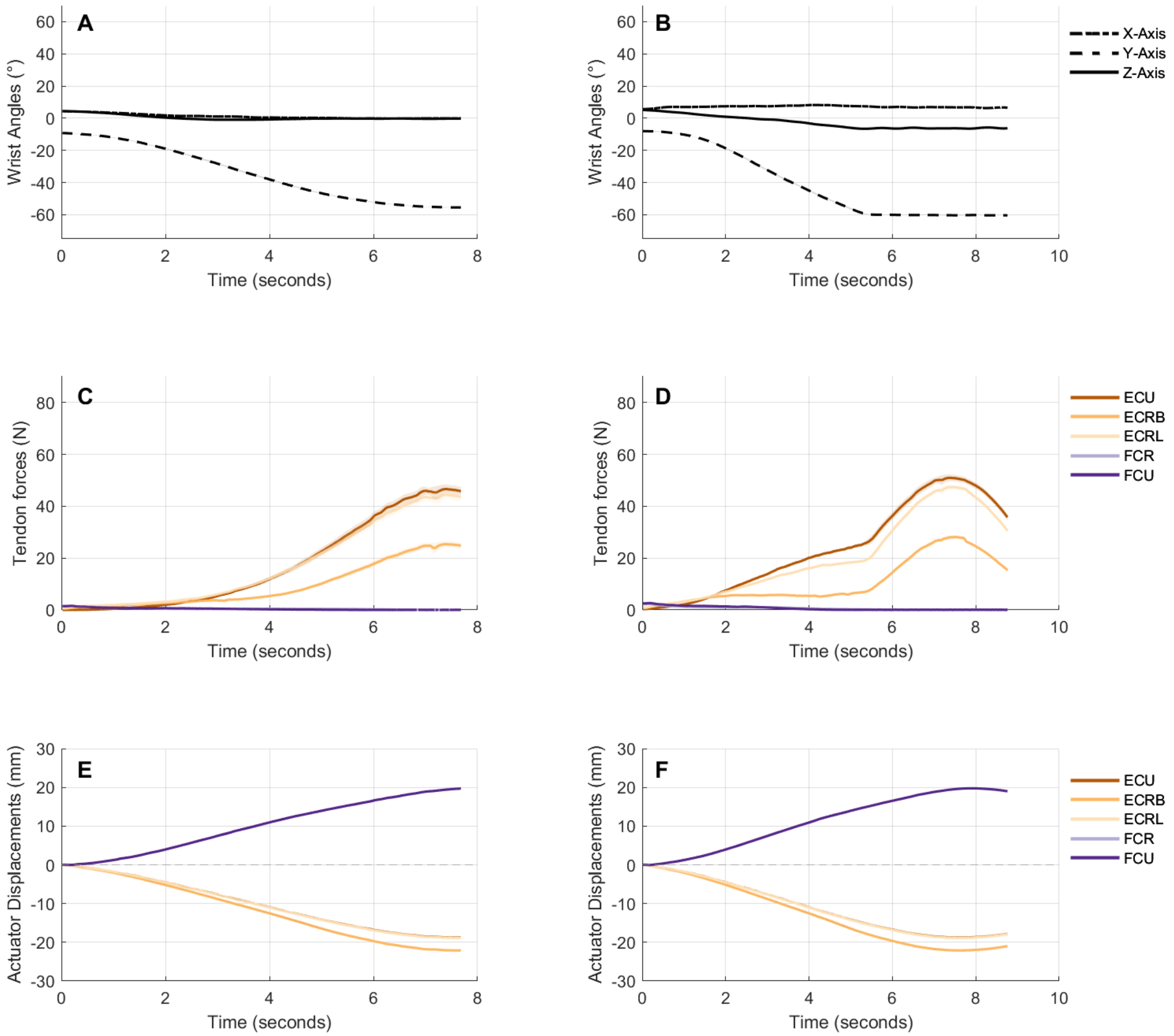

Figure S28: Average extension trial of specimen 5.

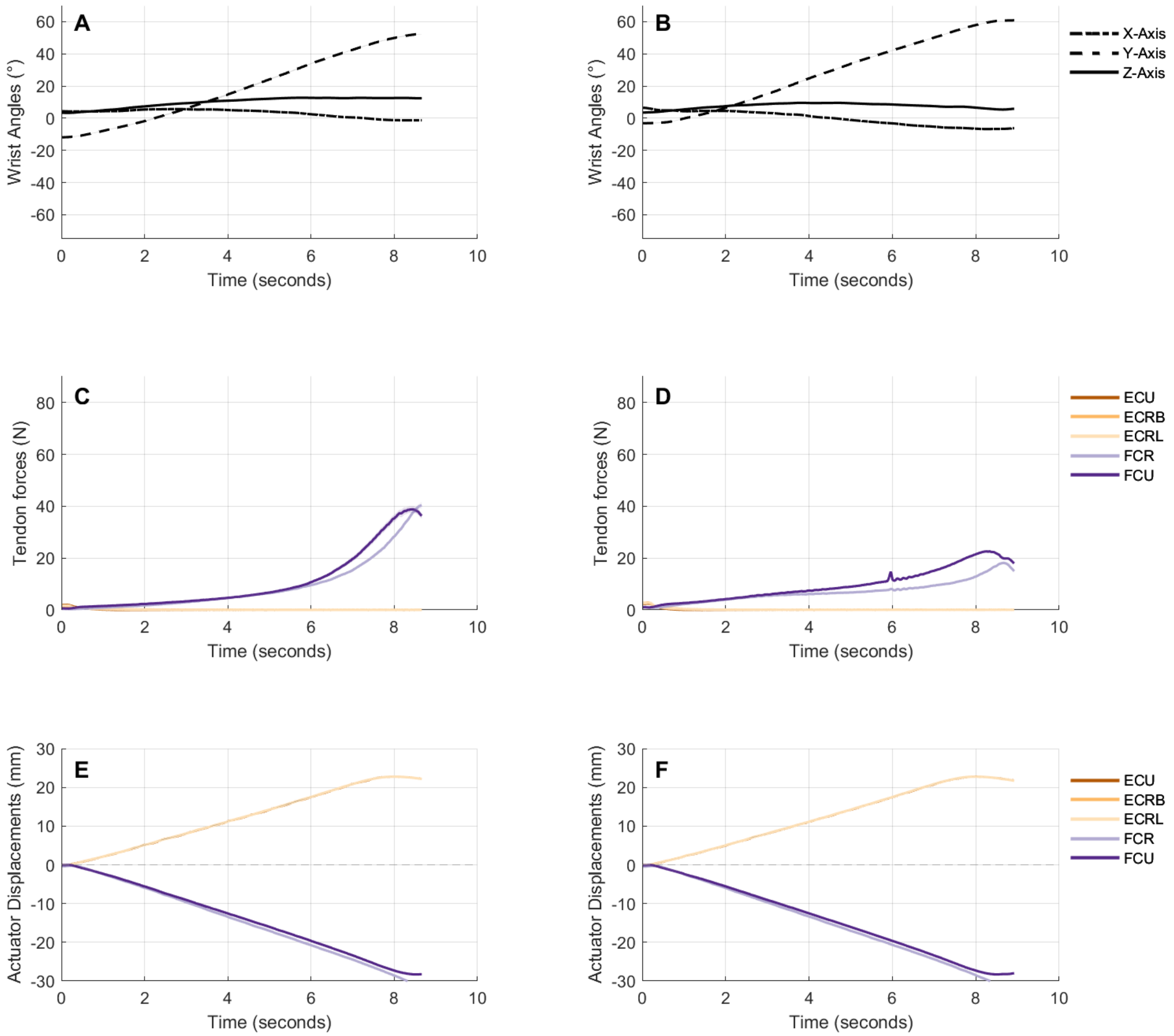

Figure S29: Average flexion trial of specimen 5.

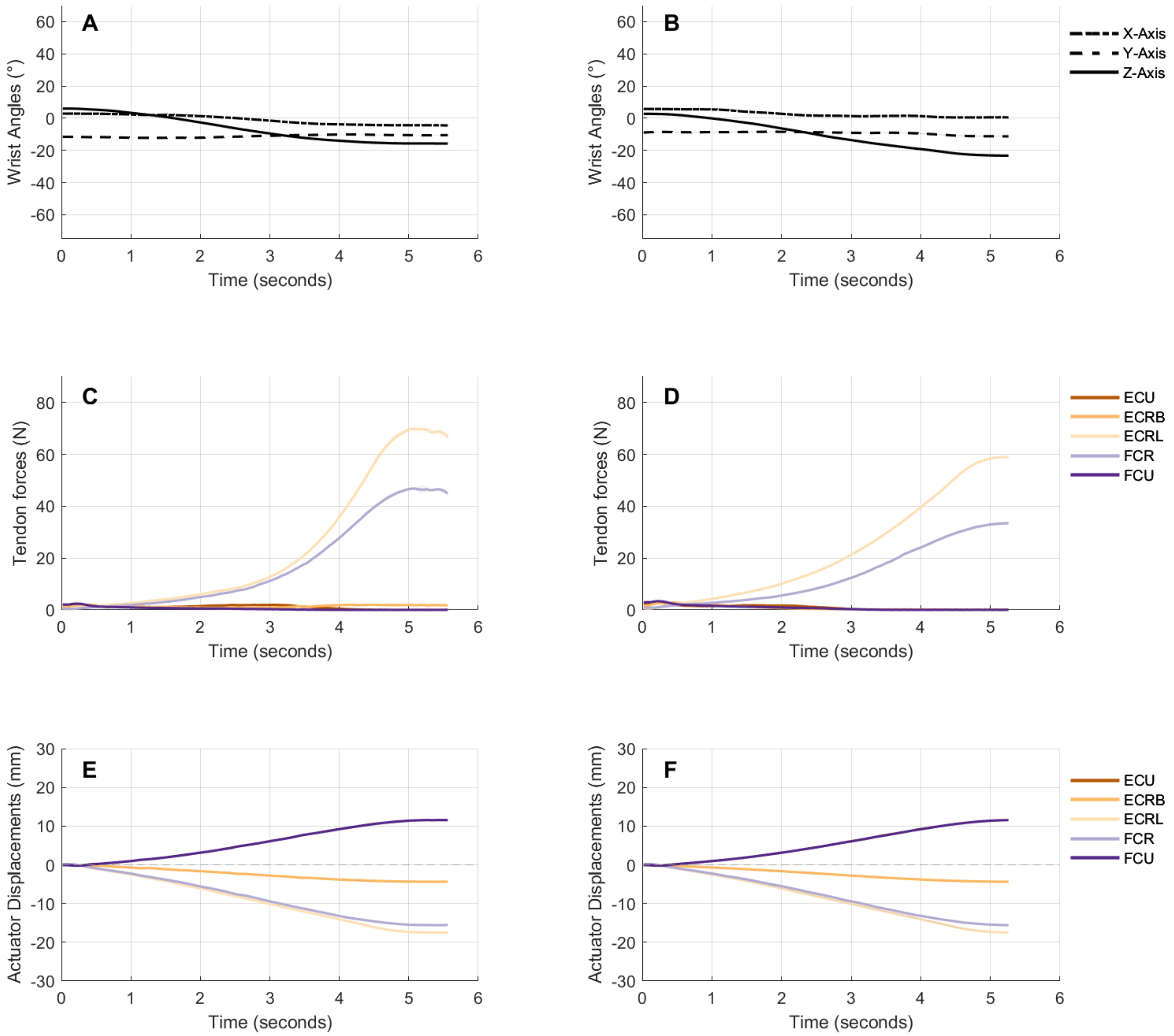

Figure S30: Average radial deviation trial of specimen 5.

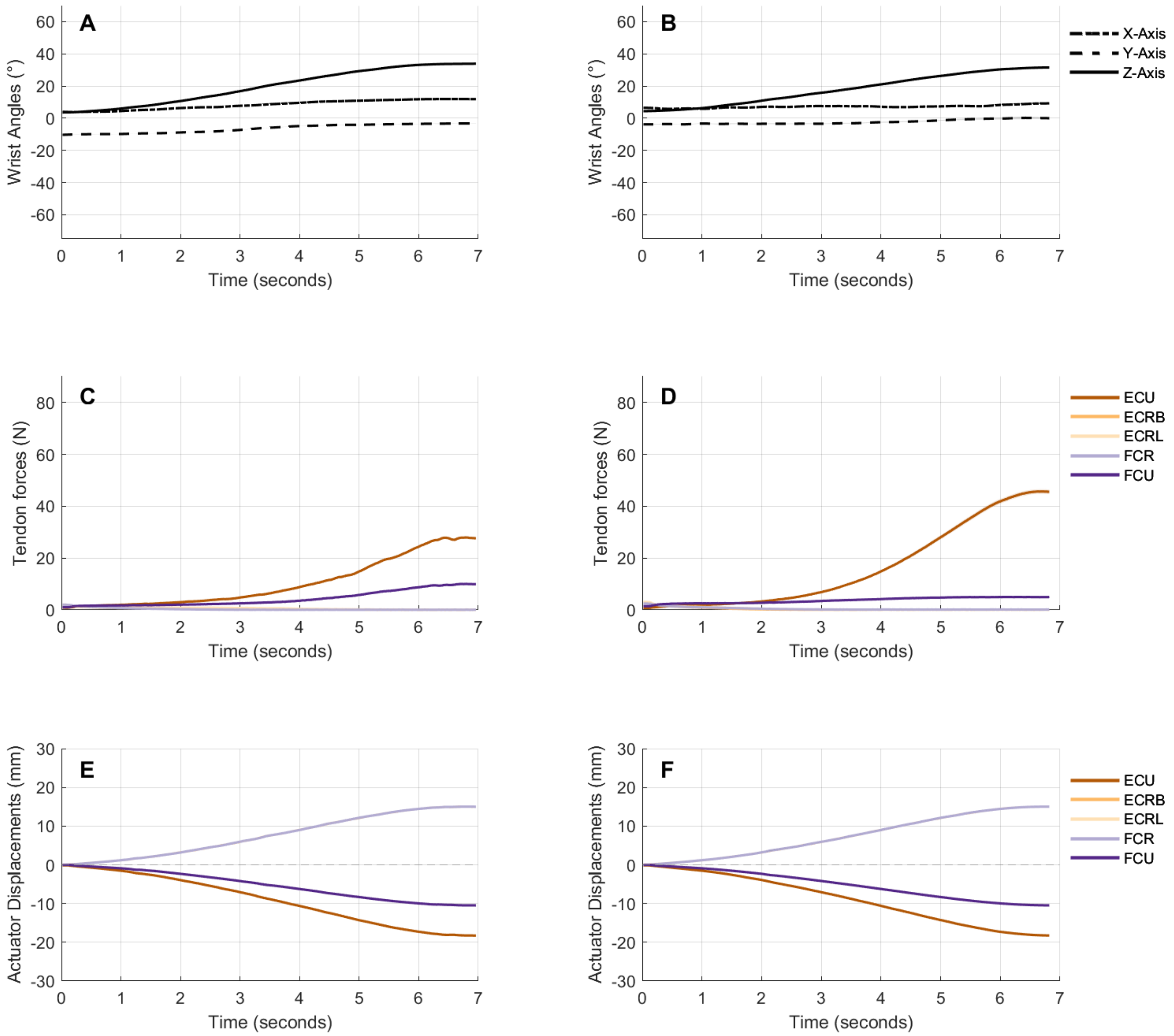

Figure S31: Average ulnar deviation trial of specimen 5.

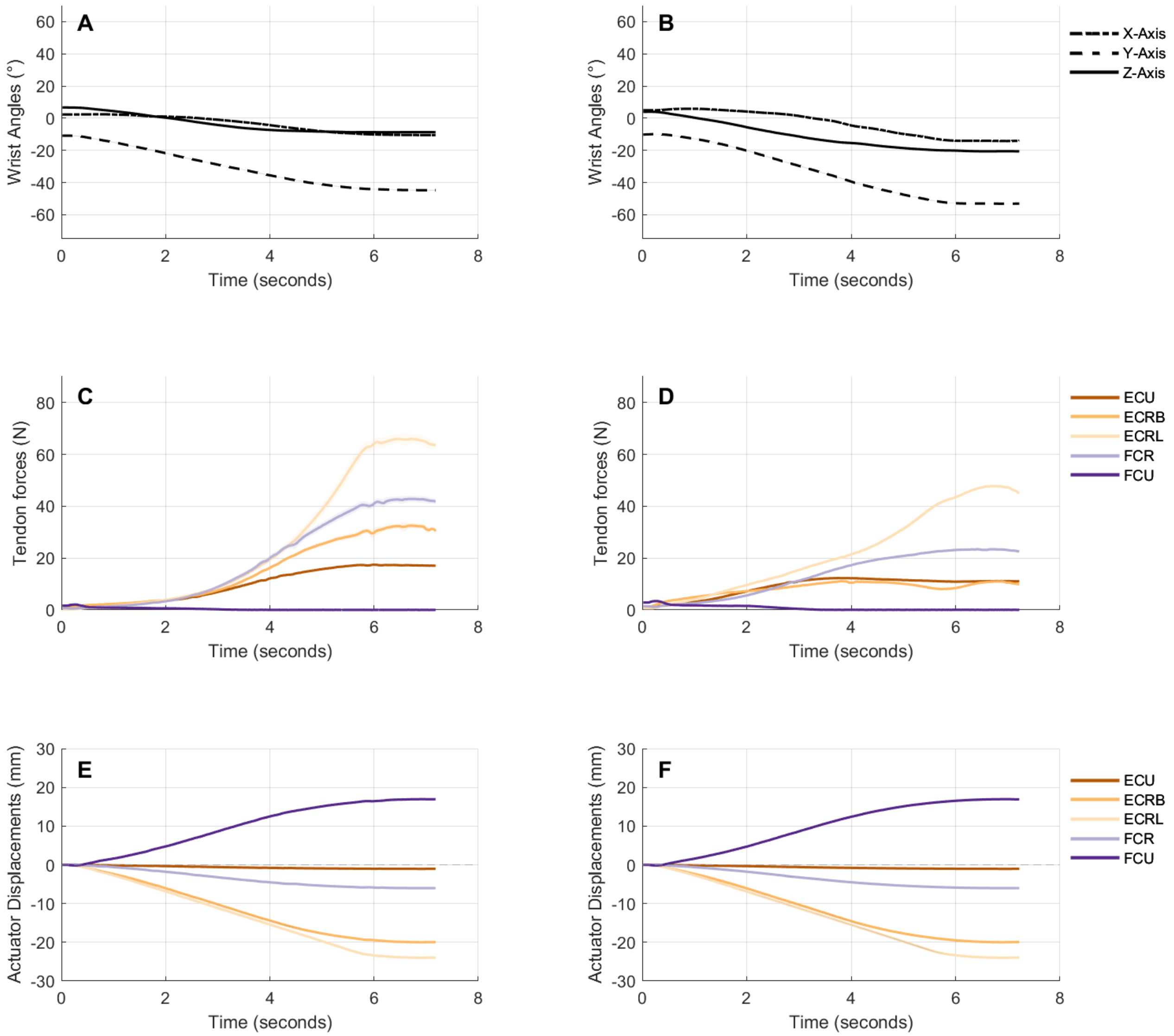

Figure S32: Average radial extension trial of specimen 5.

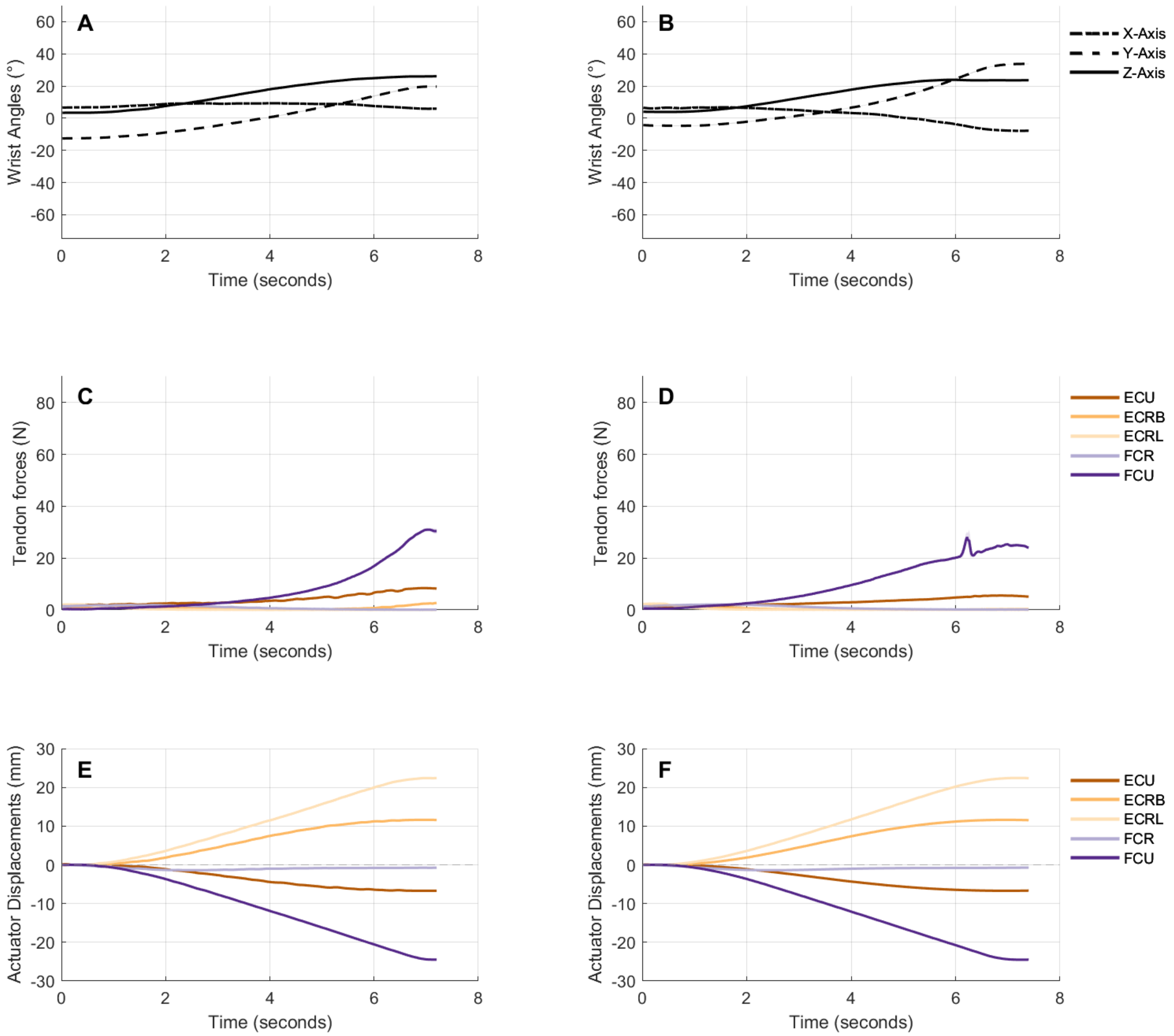

Figure S33: Average ulnar flexion trial of specimen 5.

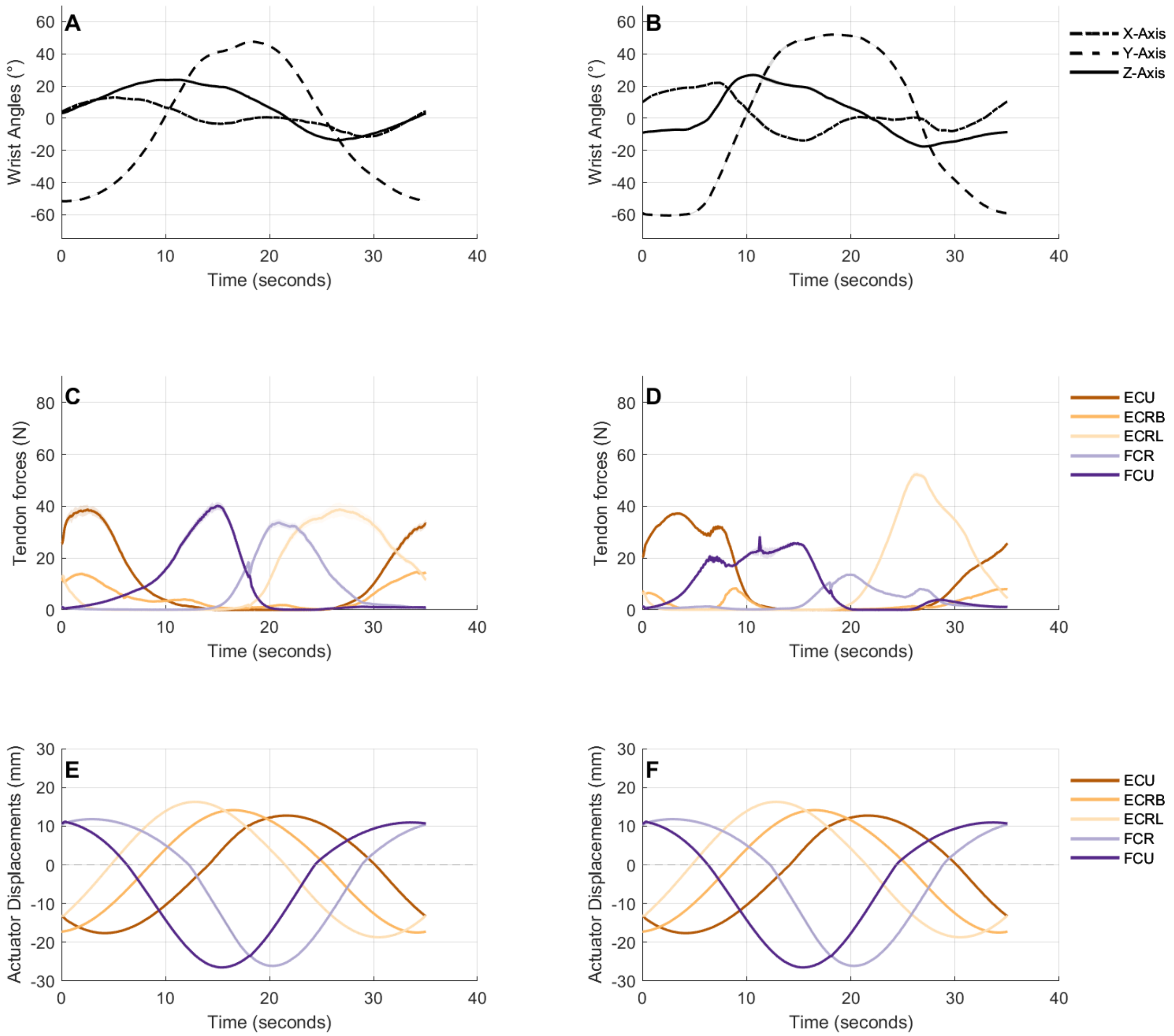

Figure S34: Average circumduction trial of specimen 5.

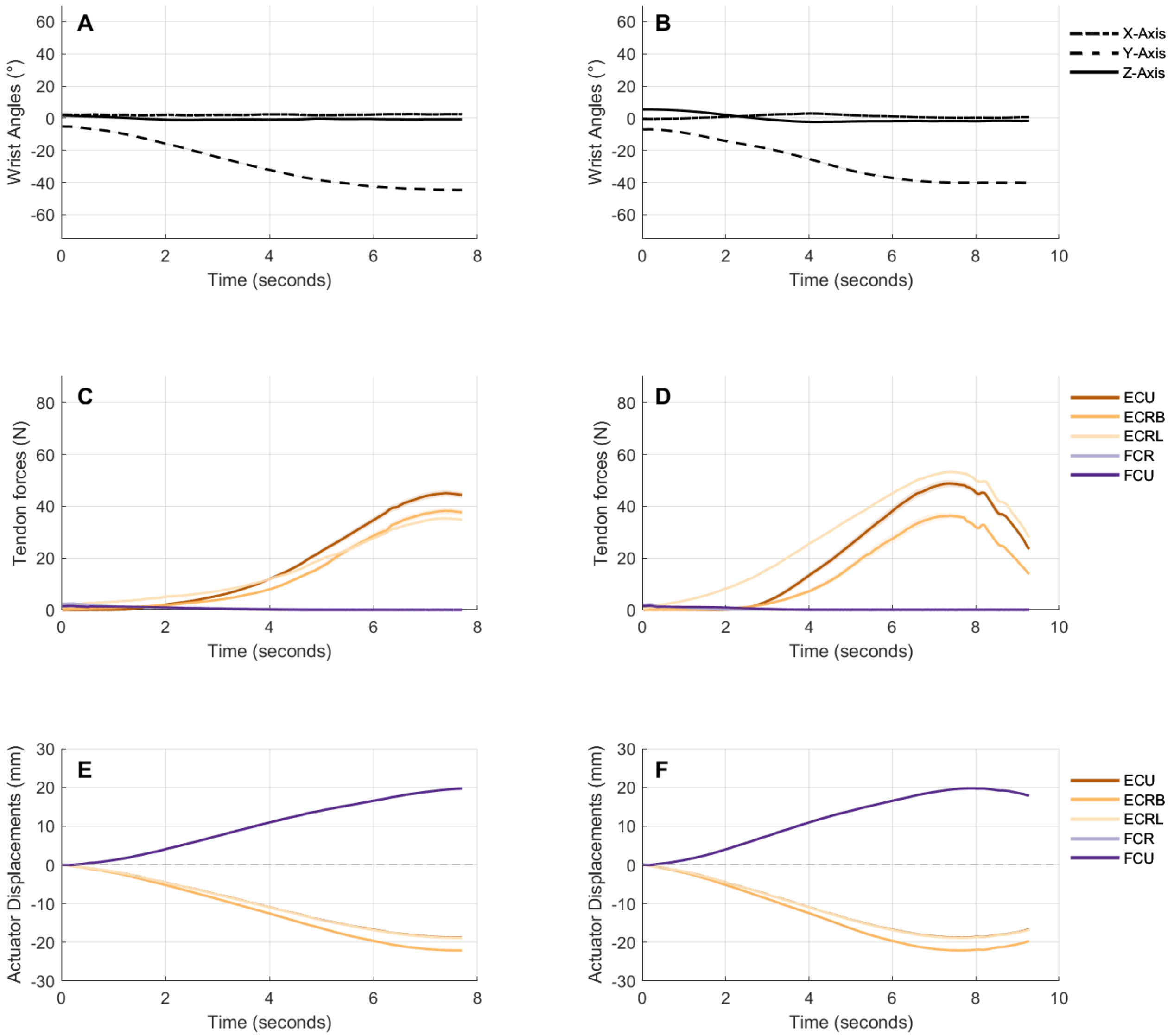

Figure S35: Average extension trial of specimen 6.

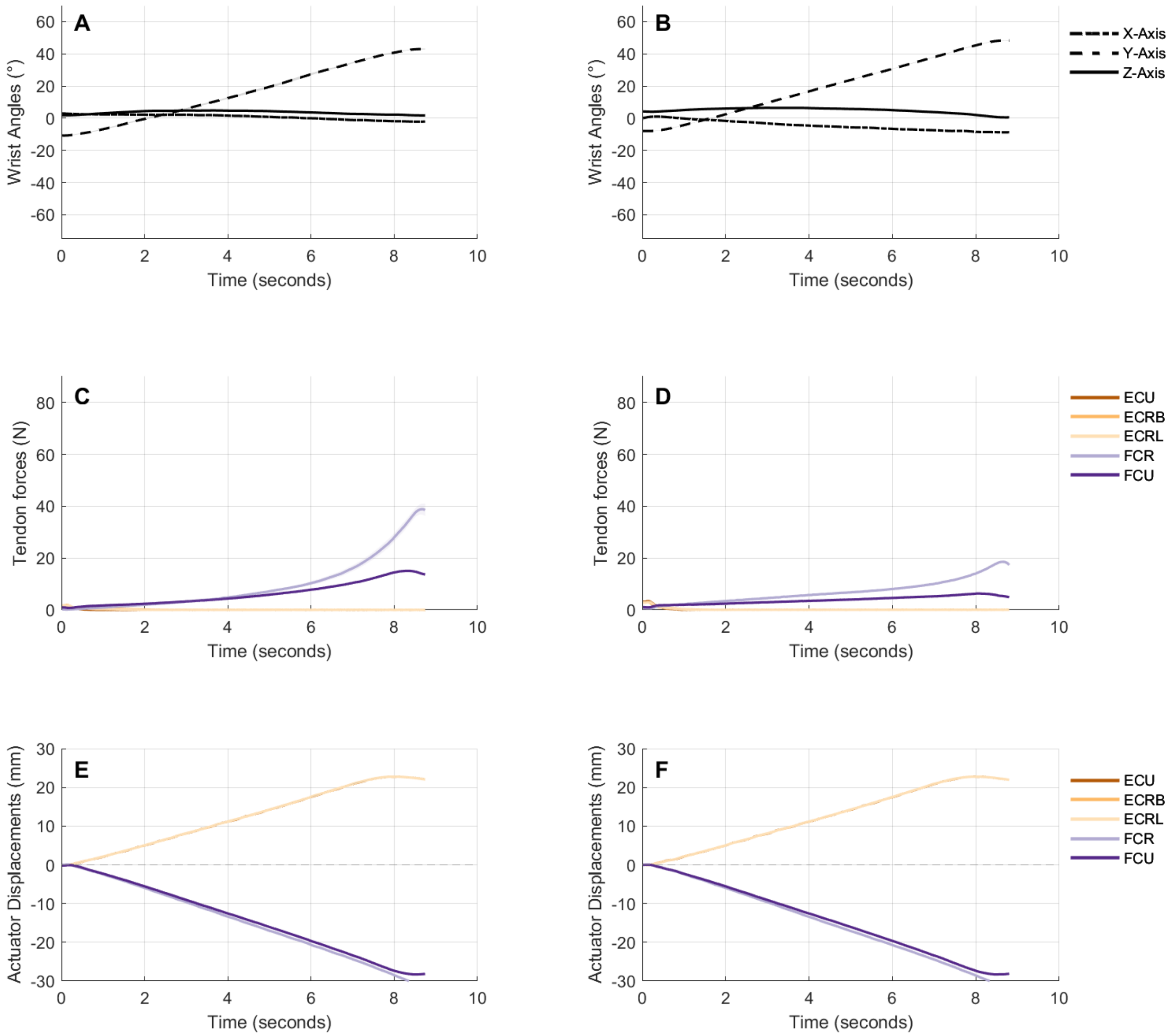

Figure S36: Average flexion trial of specimen 6.

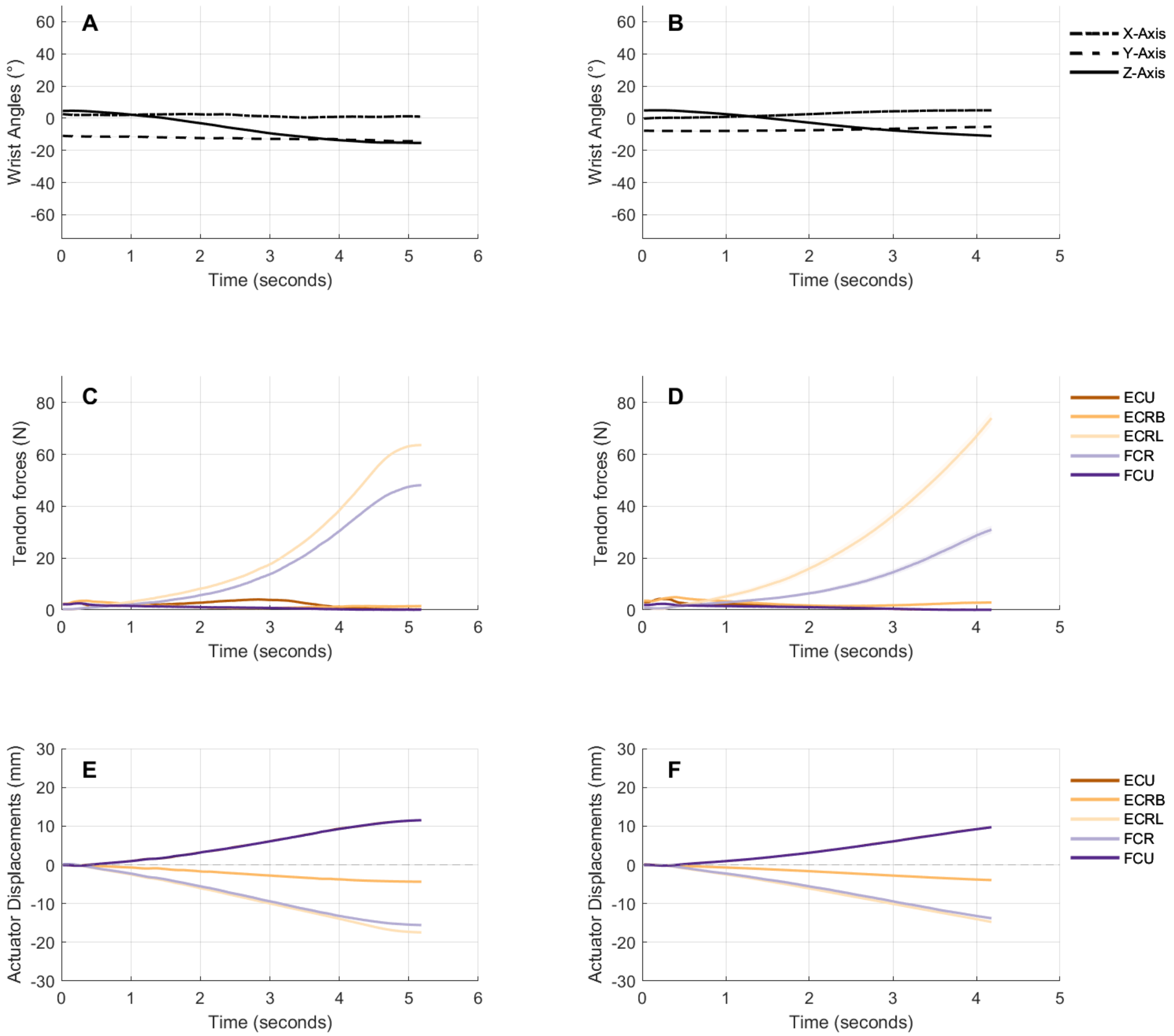

Figure S37: Average radial deviation trial of specimen 6.

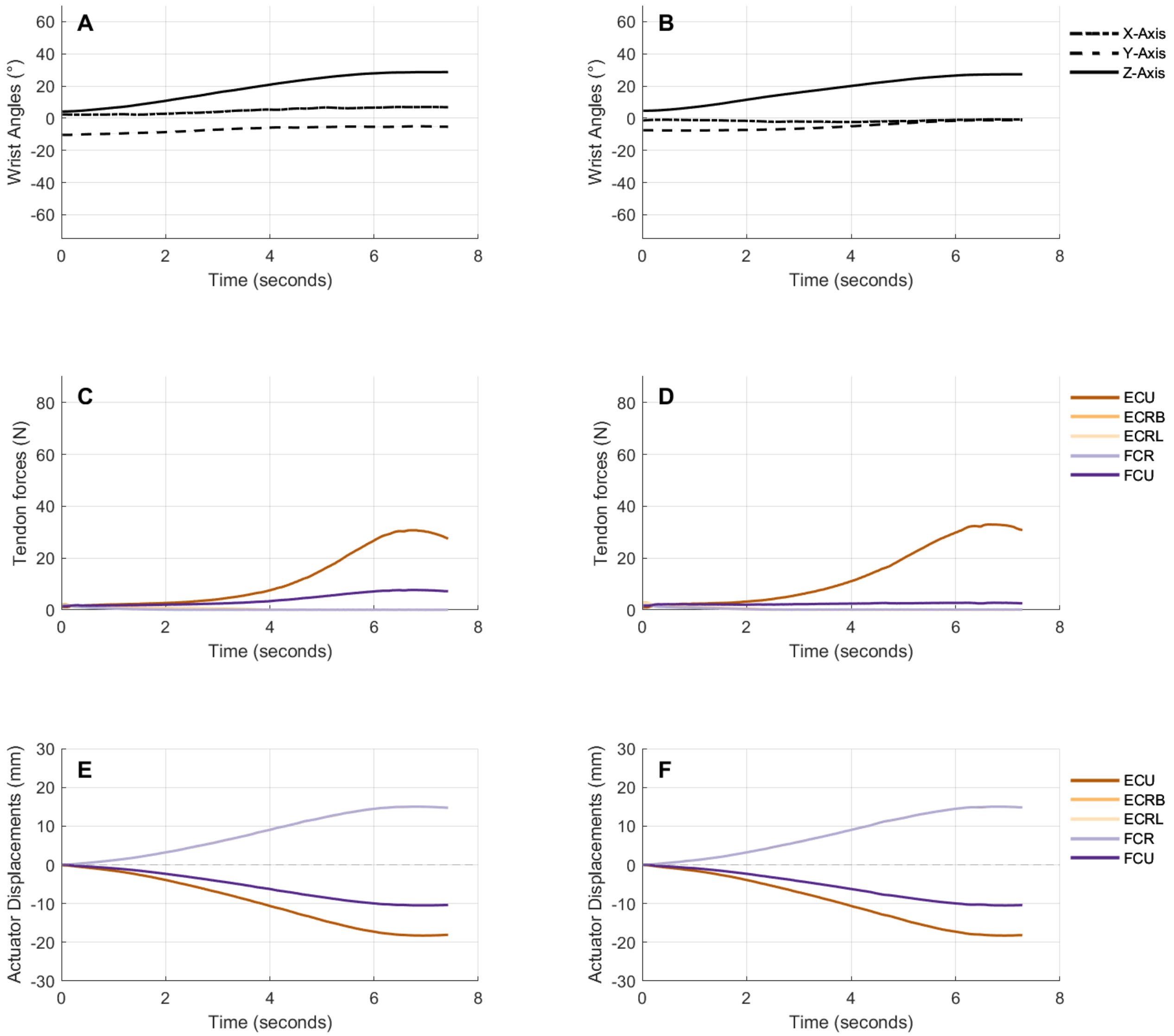

Figure S38: Average ulnar deviation trial of specimen 6.

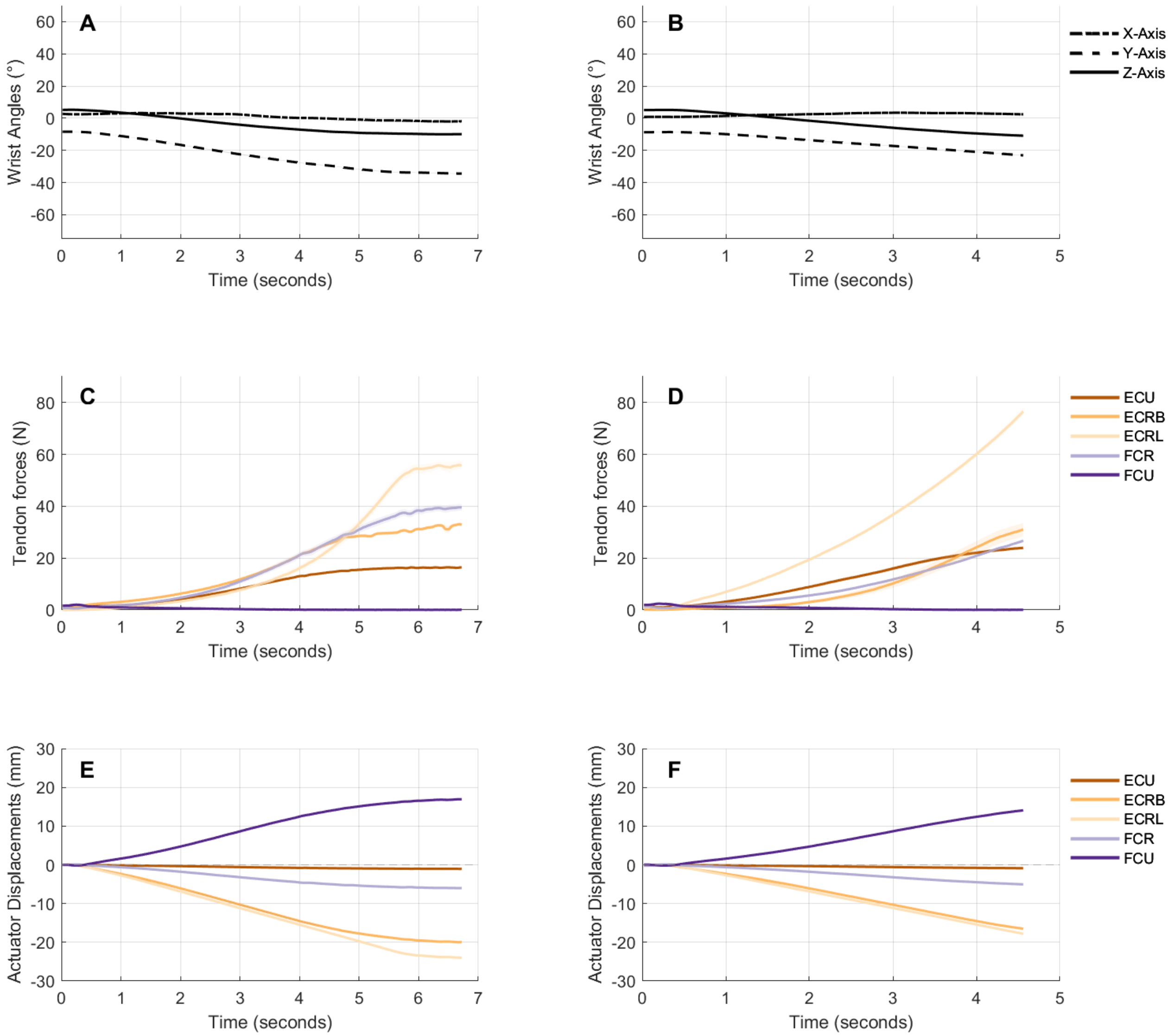

Figure S39: Average radial extension trial of specimen 6.

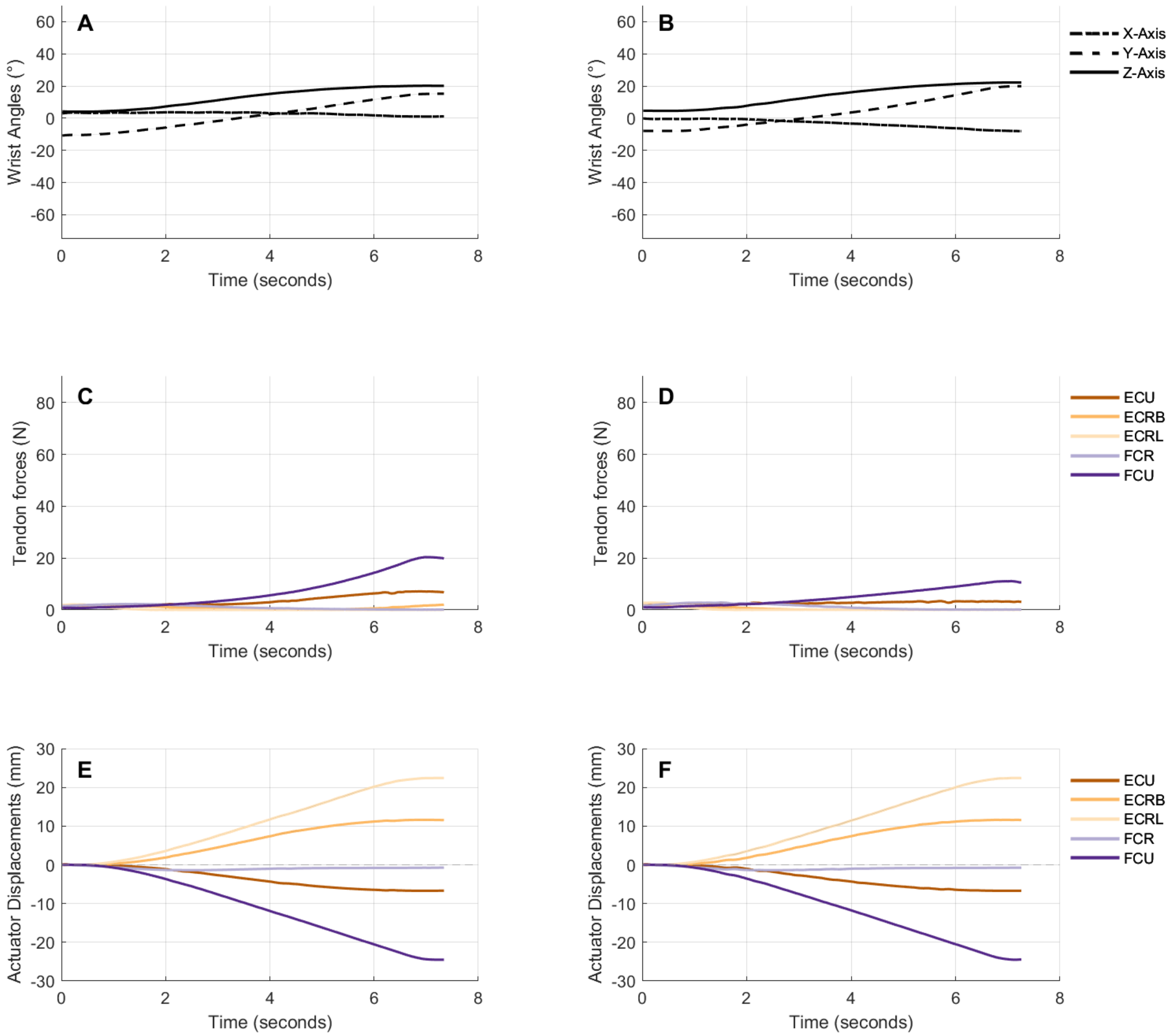

Figure S40: Average ulnar flexion trial of specimen 6.

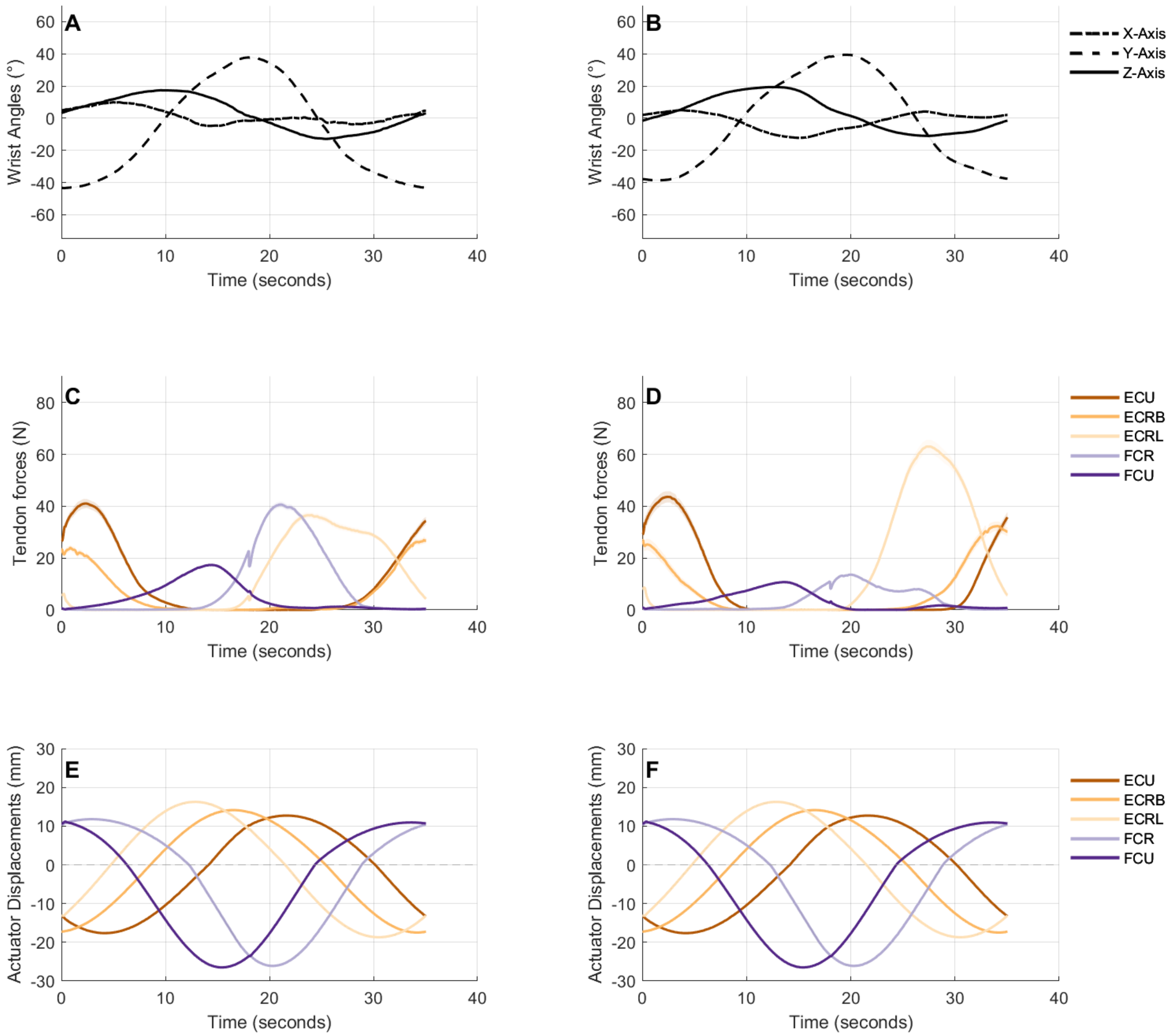

Figure S41: Average circumduction trial of specimen 6.
